# Australasian Resuscitation In Sepsis Evaluation: FLUid or vasopressors In emergency Department Sepsis (ARISE FLUIDS) trial: study protocol

**DOI:** 10.1136/bmjopen-2025-101215

**Published:** 2025-07-20

**Authors:** Belinda D Howe, Stephen P J Macdonald, Glenn Arendts, Rinaldo Bellomo, Jonathon Burcham, Anthony Delaney, Diana Egerton-Warburton, Daniel Fatovich, John F Fraser, Alisa Higgins, Peter Jones, Gerben Keijzers, Elissa Milford, Andrew Alexander Udy, Patricia Williams, Paul Young, Sandra L Peake

**Affiliations:** 1 ANZIC Research Centre, Monash University, Melbourne, Victoria, Australia; 2 Centre for Clinical Research in Emergency Medicine, Harry Perkins Institute of Medical Research, Perth, Western Australia, Australia; 3 University of Western Australia, Perth, Western Australia, Australia; 4 Medical School, The University of Western Australia, Crawley, Western Australia, Australia; 5 Emergency Medicine, Fiona Stanley Hospital, Murdoch, Western Australia, Australia; 6 Department of Intensive Care, Austin Hospital, Melbourne, Victoria, Australia; 7 Emergency Department, Royal Perth Hospital, Perth, Western Australia, Australia; 8 The George Institute for Global Health, Sydney, New South Wales, Australia; 9 Intensive Care, Royal North Shore Hospital, St Leonards, New South Wales, Australia; 10 Emergency Department, Monash Medical Centre Clayton, Clayton, Victoria, Australia; 11 Department of Medicine, School of Clinical Sciences, Monash University Faculty of Medicine Nursing and Health Sciences, Clayton, Victoria, Australia; 12 Emergency Medicine, Royal Perth Hospital, University of Western Australia and the Centre for Clinica, Perth, Western Australia, Australia; 13 Critical Care Research Group, The Prince Charles Hospital and University of Queensland, Brisbane, Queensland, Australia; 14 Adult Intensive Care Services, The Prince Charles Hospital, Brisbane, Queensland, Australia; 15 Surgery, University of Auckland, Auckland, New Zealand; 16 Adult Emergency, Auckland District Health Board, Auckland, New Zealand; 17 School of Medicine, Griffith University, Southport, Queensland, Australia; 18 Department of Emergency Medicine, Gold Coast University Hospital, Southport, Queensland, Australia; 19 Intensive Care Unit, Royal Brisbane and Women’s Hospital, Herston, Queensland, Australia; 20 ANZIC-RC, Monash University Faculty of Medicine Nursing and Health Sciences, Clayton, Victoria, Australia; 21 Department of Intensive Care, The Queen Elizabeth Hospital, Woodville South, South Australia, Australia; 22 Discipline of Acute Care Medicine, The University of Adelaide, Adelaide, South Australia, Australia; 23 Wellington Hospital, Wellington, New Zealand

**Keywords:** Sepsis, Fluid Therapy, Hemodynamics, Shock, Septic, Vasoconstrictor Agents

## Abstract

**Introduction:**

International consensus guidelines support the initial administration of 30 mL/kg of intravenous fluids for haemodynamic resuscitation of newly diagnosed septic shock. Practice variation exists between the volume of fluids administered and timing of vasopressor commencement. The optimal approach in patients with septic shock is uncertain.

**Methods and analysis:**

Australasian Resuscitation In Sepsis Evaluation: FLUid or vasopressors In emergency Department Sepsis is a 1000-participant multicentre, randomised, open-label, parallel group clinical trial conducted in patients with septic shock presenting to the emergency department in participating sites in Australia, New Zealand and Ireland. Participants are randomised (1:1) to either restricted fluids and early vasopressors or a larger initial intravenous fluid volume and later vasopressors. The primary outcome is days alive and out of hospital at day 90 postrandomisation. Secondary outcomes are all-cause mortality at day 90, time from randomisation until death (to day 90), days alive and at home at day 90 and ventilator-free, vasopressor-free and renal replacement-free days to day 28 postrandomisation and death or disability at 6-month and 12-month postrandomisation. Health-related quality of life will be assessed at day 180 and 12 months following randomisation.

**Ethics and dissemination:**

The study was approved by Northern Sydney Local Health District Human Research Ethics Committee (HREC2020/ETH02874) on 21 January 2021. Patients will be enrolled under a waiver of prior consent. The patient or next-of-kin (or equivalent according to local jurisdiction) is approached at the first available opportunity and given a trial information sheet. According to local approvals, the patient or next-of-kin chooses to either continue in the trial or opt-out/decline continued participation. Results will be disseminated in peer-reviewed journals and presented at academic conferences.

**Trial registration number:**

NCT04569942

**STRENGTHS AND LIMITATIONS OF THIS STUDY**

- Australasian Resuscitation In Sepsis Evaluation: FLUid or vasopressors In emergency Department Sepsis is a parallel group, open-label, randomised clinical trial evaluating initial fluid and vasopressor administration in early septic shock.
- This multicentre study is being conducted in the emergency department and intensive care units of 47 hospitals across three countries.
- The primary outcome, days alive out of hospital to day 90, is consumer-informed, patient-centred and captures both death and the burden of disease.
- The intervention is not blinded, which may introduce bias. However, allocation concealment is maintained until after randomisation and the primary outcome is objective and not susceptible to ascertainment bias.
- The results will be generalisable and inform international guidelines for initial haemodynamic resuscitation in septic shock.

## Introduction

 Septic shock is a medical emergency requiring urgent treatment to restore adequate perfusion to the tissues, along with measures to control the underlying infection. The Surviving Sepsis Campaign (SSC) guidelines recommend at least 30 mL/kg of intravenous fluid within the first 3 hours as first-line therapy for septic shock with subsequent addition of vasoactive agents for persistent refractory shock despite adequate fluid resuscitation.^1^ Of note, the quality of evidence guiding initial resuscitation is low and the recommendation was downgraded to weak in the 2021 guidelines. This lack of robust data to guide fluid administration and timing of vasopressor commencement has led to practice variation in the management of patients presenting to the emergency department (ED) with early septic shock.^2^

Observational studies and a systematic review have suggested that higher volumes of intravenous fluids and/or later vasopressors are associated with worse clinical outcomes, including mortality, ventilation and duration of stay.^3^,^6^ In Scandinavia, a large randomised trial of restrictive fluids versus standard care for intensive care patients with septic shock (CLASSIC) found no mortality benefit at 90 days.^7^ More recently, a large, multicentre, randomised trial conducted in the USA (CLOVERS) evaluated restricted fluids and early vasopressor strategy for patients presenting to the ED with septic shock. CLOVERS planned to recruit 2320 patients but was stopped early for futility at the second interim analysis after 1563 patients were recruited with no mortality benefit demonstrated.^8^

Given the paucity of high-quality evidence to guide clinicians on the initial resuscitation of patients with septic shock, we designed the Australasian Resuscitation In Sepsis Evaluation: FLUid or vasopressors In emergency Department Sepsis (ARISE FLUIDS) multicentre, randomised, open-label, parallel group clinical trial to determine if a strategy of restricted IV fluid volume and earlier introduction of vasopressors (vasopressors) compared with a strategy which involves a larger initial IV fluid volume and later introduction of vasopressors if required (fluids) to restore systemic arterial blood pressure, for haemodynamic resuscitation of patients with early septic shock presenting to the ED improves outcomes, including days alive out of hospital to day 90 (DAOH-90). This paper describes the ARISE FLUIDS trial protocol and follows the Standard Protocol Items: Recommendations for Interventional Trials Standard Protocol Items: Recommendations for Interventional Trials 2013 statement.^9^ A separate statistical analysis plan paper will also be submitted for publication prior to completing patient recruitment.

## Methods and analysis

### Trial management

ARISE FLUIDS is registered on ClinicalTrials.gov (NCT 04569942) and is endorsed by the Australian and New Zealand Intensive Care Society (ANZICS) Clinical Trials Group (CTG) and the Australasian College for Emergency Medicine (ACEM) Clinical Trials Network (CTN). The global trial sponsor is Monash University, Melbourne, Australia. The trial is coordinated by the Australian and New Zealand Intensive Care Research Centre (ANZIC-RC), Monash University. The ANZIC-RC provides overall trial oversight of all trial activities and will undertake statistical analyses of study data. Additionally, it will coordinate monitoring of data collection, ethics, regulatory and legal approvals in Australia and Ireland. The Medical Research Institute of New Zealand, Wellington is the local sponsor in New Zealand (NZ) overseeing trial conduct at NZ trial sites including monitoring of data collection, ethics, regulatory and legal approvals. Trial management is overseen by a management committee (online supplemental Appendix 1) with frequent oversight by the Working Party of the management committee (online supplemental Appendix 2).

### Patient and public involvement

Patients with a lived experience of septic shock were involved in the design and conduct of this research. Two patients are included on the trial management committee and have provided input on the choice of outcomes and consent models.

### Protocol version

This document is based on information contained in the study protocol V.3, dated 10 October 2024 (online supplemental Appendix 3) and registered on ClinicalTrials.gov (NCT 04569942).

### Trial design and participants

ARISE FLUIDS is a multicentre, randomised, open-label, parallel group clinical trial that will recruit 1000 patients presenting with septic shock to the ED of participating hospitals. The primary outcome is DAOH-D90.

Recruitment commenced in October 2021 and currently 47 hospitals in Australia, New Zealand and Ireland are actively recruiting. Target recruitment is anticipated in mid to late 2025 with completion of data collection in late 2026 (for 12-month follow-up). Eligible patients are 18 years or older and presenting to the ED of a participating institution with septic shock. Full inclusion and exclusion criteria are listed in table 1. Eligible patients must meet all the inclusion criteria within the first 6 hours of ED presentation and must be randomised within 2 hours of meeting the final inclusion criterion.

**Table 1:** Patient eligibility criteriae

Inclusion criteria	Clinically suspected infection
	SBP <90 mm Hg or MAP <65 mm Hg, despite ≥1000 mL total intravenous fluid bolus(es) administered over ≤60 minutes, including pre-hospital fluid boluses*
	Arterial or venous blood lactate >2.0 mmol/L
	At least one dose of an intravenous antimicrobial commenced
Exclusion criteria	Age<18 years
	Confirmed or suspected pregnancy
	Transferred from another acute care facility
	Hypotension suspected to be due to a non-sepsis cause
	>2000 mL total intravenous fluid administered, including prehospital fluids†
	>6 hrs elapsed since ED presentation‡>2 hrs elapsed since final inclusion criterion met
	Treating clinician considers one or both treatment regimens not suitable for the patient or study protocol cannot be delivered, eg, limitation of care, requirement for immediate surgery
	Death considered imminent or inevitable
	Underlying disease makes survival to 90 days unlikely
	Inability to follow patient up to 90 days, eg, overseas visitor
	Previously enrolled in ARISE FLUIDS

* A bolus is the rapid administration of intravenous fluid for haemodynamic resuscitation, including prehospital boluses given in the ambulance. A total of ≥1000 mL of intravenous fluid may consist of 500–1000 mL boluses. Each bolus needs to be given over ≤ 60 minutes. Bolus fluid does not include maintenance fluids and the type of resuscitation fluid administered is not specified.

† Total intravenous fluid administration excludes drugs and flushes.

‡ Defined as ED triage time.

ARISE FLUIDS, Australasian Resuscitation In Sepsis Evaluation: FLUid or vasopressors In emergency Department Sepsis; ED, emergency department; hrs, hours; MAP, mean arterial pressure; SBP, systolic blood pressure.

### Randomisation and blinding

Eligible patients are randomised in a 1:1 ratio using permuted block randomisation with variable block sizes, stratified by site to receive either restricted fluids with early vasopressor administration (*vasopressor arm*) or a larger initial volume of fluid resuscitation followed by later vasopressor administration (*fluids arm*), if required. Allocation concealment is maintained via central randomisation and performed by trained local research or clinical staff using a secure, password-protected, web-based database available 24 hours a day, 7 days a week. The nature of the study interventions means that blinding to the allocated treatment is not possible. Bias is minimised by ensuring allocation concealment until after randomisation, regular monitoring of compliance with the study protocol and an objective primary outcome that is not susceptible to ascertainment bias.

### Study interventions

**Figure 1:**
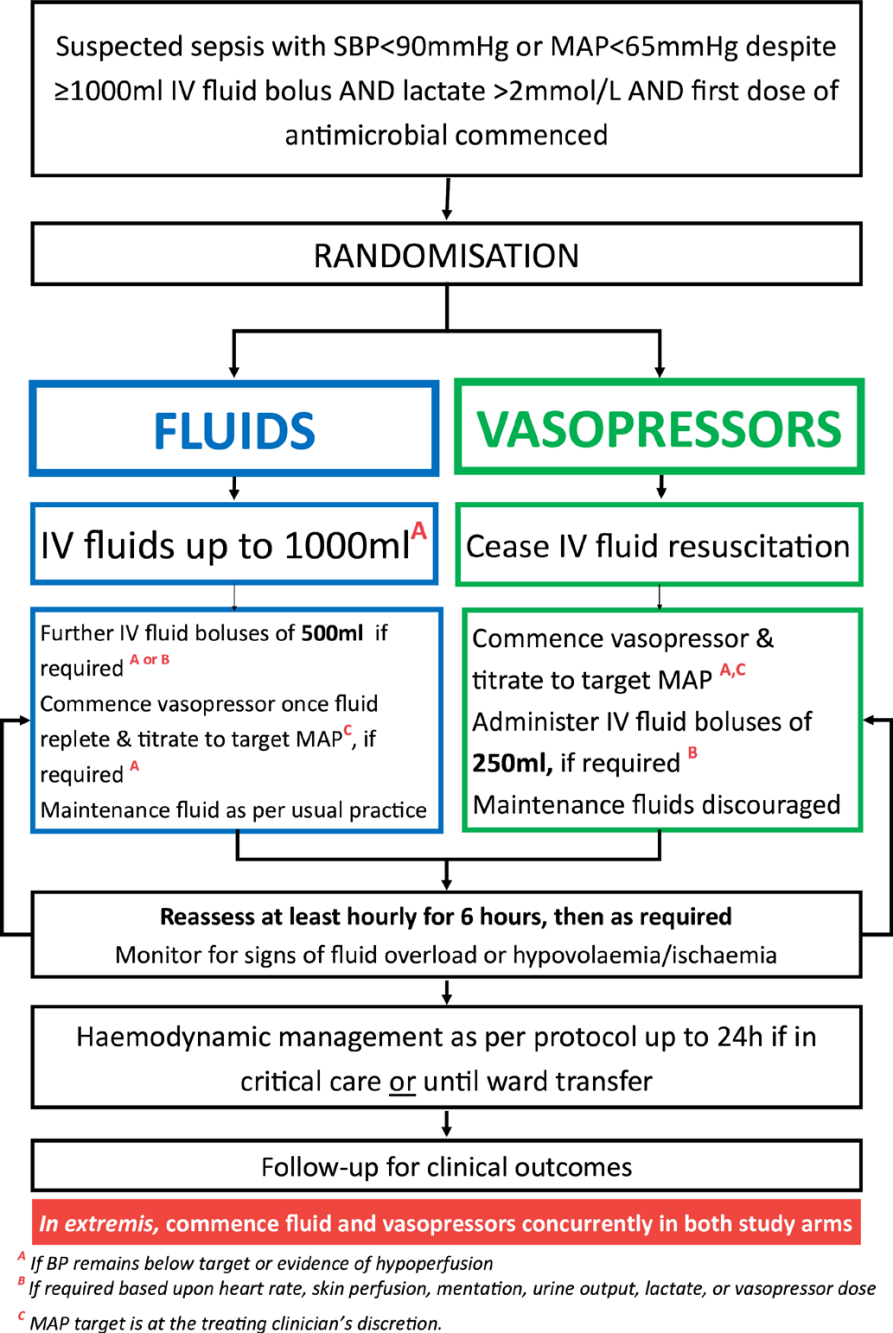
ARISE FLUIDS study algorithm. Boluses are defined as the administration of 500–1000 mL intravenous fluid with each bolus administered over 60 min or less. Bolus fluid does not include maintenance fluid. In the fluids arm, a minimum of 2–3 L (30 mL/kg) is recommended within 3 hours of arrival to the ED and includes pre-hospital fluid boluses. The type of resuscitation fluid (crystalloid, colloid or blood product), vasopressor and route of vasopressor administration (central venous or peripheral) is at the discretion of the treating clinician. The intervention is delivered for a minimum of 6 hours and up to 24 hours while the patient remains in a critical care area (ED, ICU, HDU). All other aspects of care are according to the treating clinician. ARISE FLUIDS, Australasian Resuscitation In Sepsis Evaluation: FLUid or vasopressors In emergency Department Sepsis; ED, emergency department; HDU, high dependency unit; ICU, intensive care unit; MAP, mean arterial pressure; SBP, systolic blood pressure.

Following randomisation to the vasopressor arm, intravenous fluid resuscitation will be immediately ceased and a vasopressor infusion titrated to achieve a target mean arterial pressure (MAP). The choice of vasopressor and the target MAP will be determined by the treating clinical team with participants to be reassessed at least hourly for 6 hours postrandomisation, then as clinically required. Intravenous fluid boluses of 250 mL will be permitted if clinically indicated with the assessment of fluid status determined by the treating clinician according to usual local practice. This includes evidence of refractory hypotension, delayed capillary refill time, lactate >4 mmol/L or rising from previous level despite at least 2 hours of resuscitation, persistent tachycardia or oliguria <0.5 mL/kg/hour for at least 2 hours. Maintenance fluids are strongly discouraged (figure 1).

Following randomisation to the fluids arm, an intravenous fluid bolus of up to 1000 mL will be administered over a maximum of 1 hour for persisting hypotension or hypoperfusion. Thereafter, additional 500 mL intravenous fluid boluses will be administered to achieve the treating clinician-determined target MAP with participants to be reassessed at least hourly for 6 hours postrandomisation, then as clinically required. Fluid resuscitation will be guided by local practice and usual clinical assessment of fluid status. Consistent with the 2021 SSC guidelines for the initial resuscitation of sepsis-induced hypotension, a minimum of 2–3 L (30 mL/kg), including prerandomisation fluids, is recommended within 3 hours of arrival to the ED.^1^ A vasopressor infusion will be commenced if blood pressure remains below target once the treating clinician considers fluid resuscitation to be optimal, and the patient is not fluid-responsive. Maintenance fluids will be permitted according to local practice.

In both trial arms, the type of fluid and vasopressor administered will be determined by the treating clinical team within the protocol-guided parameters. Peripherally administered intravenous vasopressors will be permitted for up to 24 hours in accordance with local practice. The intervention will be delivered for a minimum of 6 hours, with reassessment of haemodynamic status at least hourly for the first 6 hours. The intervention will be continued to 24 hours postrandomisation while the patient is present in a critical care area (ED, intensive care unit (ICU), high dependency unit (HDU)). Transfer to ICU, HDU or the general ward from the ED and all other aspects of care, including source control, antimicrobial therapy and inotrope administration will be at the treating clinician’s discretion. At 24 hours postrandomisation, or discharge to the general ward, whichever occurs sooner, the intervention will be ceased and usual care is delivered according to the treating clinical team.

In both treatment groups, vasopressors may be commenced concurrently with fluids if the patient is *in extremis*.

### Data collection and management

Data collection includes patient screening, eligibility criteria at randomisation and baseline patient characteristics, including the volume of fluid administered prerandomisation. Physiological and laboratory data, where available as part of usual care, will be collected up to 24 hours postrandomisation. Details on fluid (type, volume) and vasopressor (type, dose, route) administration during the intervention period and daily fluid balance for the first 7 days postrandomisation while the patient remains in the ED, HDU, ICU will be recorded. If discharge to the ward occurs before the 24-hour intervention period is complete, details on fluid administration in the general ward will not be collected. Microbiology details (antimicrobial administration, microbiological organisms) will be collected up to 72 hours from ED presentation. Use and duration of organ support (mechanical ventilation, vasopressors, renal replacement therapy) cointerventions (eg, source control), complications potentially related to the study interventions (eg, acute pulmonary oedema, intestinal ischaemia) and adverse events that require significant intervention and are deemed to be causally related to the study intervention arms (possibly, probably or definitely) will be collected. Patients will be followed up until death or 12 months postrandomisation whichever occurs first. Details on all readmissions to a care facility (acute, rehabilitation, long-term) up to 90 days postrandomisation will be collected using participant self-reported diaries and medical chart review. At 6-month and 12-month postrandomisation vital status, quality of life using the European Quality of Life 5 Dimensions 5 Level questionnaire and functional status using the WHO Disability Assessment Schedule V.2.0 questionnaire will be collected by the site via telephone or post.^10^,^12^ Full information on data collection is listed in online supplemental Appendix 4.

Data will be entered into a secure web-based database by trained staff at each participating site. Data management will be coordinated by the ANZIC-RC and Medical Research Institute of New Zealand project managers, including programming and data management support (source data verification, database questions, technical issues, data queries, query resolution) according to a prespecified data monitoring plan.

### Outcomes

The primary outcome is the number of days alive and out of hospital to 90 days postrandomisation. DAOH-D90 is a clinically important and patient-centred outcome that captures both death and the burden of disease. In patients with sepsis, health-related quality of life at 6 months has been shown to be associated with DAOH-D90.^13^ The choice of primary outcome for ARISE FLUIDS has been guided by consumer input, two sepsis survivors, with time spent out of hospital and at home considered an important outcome. A primary outcome of 90-day mortality was not feasible based on our prospective observational study of 591 patients presenting to the ED of 70 Australian and New Zealand hospitals, in which hospital mortality was 6.3%.^2^ Table 2 lists the secondary and tertiary study outcomes. Health-related quality of life and functional assessments will be conducted at 6-month and 12-month postrandomisation by the site via telephone or post.

**Table 2:** Study outcomes

Primary outcome	Number of days alive and out of hospital to day 90 postrandomisation*
Secondary outcomes	All-cause mortality to day 90 postrandomisation
	Time from randomisation until death (to day 90)
	Days alive and at home to day 90 postrandomisation†
	Ventilator-free days to day 28 postrandomisation‡
	Vasopressor-free days to day 28 postrandomisation‡
	Renal replacement therapy-free days to day 28 postrandomisation‡
	Death or disability at 6-month and 12-month postrandomisation§
Tertiary outcomes	Incidence and duration of invasive mechanical ventilation
	Incidence and duration of vasopressor support
	Incidence and duration of acute renal replacement therapy
	ED duration of stay
	ICU duration of stay
	Hospital duration of stay
	In-hospital mortality (censored at 90 days)
	Mortality to 6-month and 12-month postrandomisation§
	Quality of life at 6-month and 12-month post-randomisation§
	Cost-effectiveness at 12 months measured as cost/QALY§

* For each patient, days alive and out of hospital (not in the index hospitalisation, acute hospital readmission) between randomisation and day 90 will be subtracted from 90 to calculate their number of days alive and out of hospital to day 90 postrandomisation. Patients who die on or prior to day 90 will be assigned zero days alive and out of hospital.

† Days alive and at home is returned to preadmission place of residence. For each patient, days at home (not in the index hospitalisation, acute hospital readmission, inpatient rehabilitation, in a nursing home) between randomisation and day 90 will be subtracted from 90 to calculate their number of days alive and at home to day 90 postrandomisation. Patients who die on or prior to day 90 will be assigned zero days alive and at home.

‡ Patients who die on or prior to day 28 will be assigned zero organ support-free days.

§ Disability determined by the European Quality of Life 5 Dimensions 5 Level (EQ-5D-5L) questionnaire and functional status using the World Health Organisation Disability Assessment Schedule version 2.0 (WHODAS V.2.0) questionnaire. Death or moderate-to-severe disability (ie, WHODAS score ≥25%); may be reported separately when 12-month follow-up complete.

ED, emergency department; ICU, intensive care unit; QALY, quality-adjusted life year.

### Protocol deviations and adverse events

The study will be monitored both centrally and on-site for patient eligibility, protocol compliance, data queries and safety reporting according to the study’s risk-based monitoring plan. Protocol deviations include: (1) randomisation of ineligible patients; (2) fluid arm, one or more boluses of 500 mL not administered for hypotension and (3) vasopressor arm, vasopressors not commenced for hypotension and/or hypoperfusion, fluid boluses administered without a study-specific indication and fluid boluses of greater than 250 mL administered. Adverse events will be collected from randomisation until index hospital discharge. Reporting of adverse events will be restricted to events that are deemed by the site investigator to be of concern or related to the study or the intervention arms (possibly, probably or definitely). Adverse events already defined as study complication outcomes (eg, central venous catheter complications, acute pulmonary oedema, ischaemia) will be collected in both intervention groups using standardised definitions and not reported separately as adverse events to facilitate unbiased reporting.

### Statistical analysis

Calculation of the sample size calculation was informed by the ARISE randomised clinical trial that recruited 1600 patients presenting to the ED, primarily in Australia and New Zealand, with early septic shock, the population of interest.^14^ In ARISE, the observed mean (SD) DAOH-D90 was 60(31) days in the control group. Assuming a 7-day increase in DAOH-D90 in the vasopressor arm, a sample size of 950 evaluable participants will have 90% power to detect this difference with a type 1 error rate of 0.05 (including a 15% inflation factor to account for the non-parametric distribution). A between-group difference of 7 days in DAOH-D90 was considered biologically plausible, consumer informed and clinically meaningful and with significant resource and cost implications. The desired trial recruitment total was inflated from 950 patients to 1000 patients to account for the small interim analysis effect and to accommodate up to 5% loss to follow-up. Previous studies by the ANZICS CTG have yielded a loss to follow-up rate of≤5%.^14^,^16^

### Data safety and monitoring committee

An independent, multidisciplinary data safety and monitoring committee (DSMC), composed of experienced researchers in intensive care and emergency medicine, clinical trials and biostatistics without other connection to ARISE FLUIDS was established prior to commencing patient recruitment to review safety and outcome analyses and serious adverse events at the predetermined single safety and efficacy interim analysis, and as deemed appropriate. Specific details of the roles and responsibilities are outlined in the DSMC charter (online supplemental Appendix 5). The interim analysis was performed on completion of follow-up to day 90 postrandomisation of the first 500 participants (50% target recruitment). Data were analysed by the unblinded study statistician according to group allocation. All data remain confidential to the DSMC and the trial statistician. Stopping rules for efficacy were based on the HayBittle-Peto boundary approach with a p value of 0.005 used to indicate benefit at the interim analysis while retaining an unmodified p value of 0.05 for the final analysis. There were no predefined stopping rules for aspects pertaining to safety and no plans to stop ARISE FLUIDS for futility.

### Analysis of populations

The final population analyses will be by intention-to-treat according to the participant’s randomly allocated group, regardless of treatment compliance. If consent for participation is withdrawn or consent to continue is not given, the data will not be used unless specific approval to do so is obtained. Initial range and logic tests will be performed, and discrepancies will be corrected with participating sites and source data where appropriate. The randomisation code will be broken after the blinded analysis of the primary and secondary outcomes is accepted by the study management committee. There will be no imputation of missing data, including for the primary outcome DAOH-D90. DAOH-D90 and other non-parametric outcomes will be analysed using quantile regression with results presented as median difference (95% CI). Mortality and other binomial outcomes will be analysed using logistic regression with results reported as ORs (95% CI). Time to event outcomes will be analysed using proportional hazards regression with results presented as Kaplan-Meier or cumulative incidence curves where mortality is a competing risk. Functional status and quality of life will employ longitudinal analysis techniques to account for data collected at both 6 and 12 months. All analyses will account for site with participants nested within site and site treated as a random effect. Where baseline imbalance (p<0.2) is observed between treatment arms, sensitivity analysis will be performed adjusting for imbalanced variables. Sensitivity to missingness will be performed using multiple imputation. A secondary analysis of the primary outcome will be conducted using a Bayesian approach to model a posterior probability of the OR for DAOH-D90 postrandomisation between the intervention and control group with associated 95% credible interval. A preplanned subgroup analysis will be performed according to the prerandomisation characteristics of age dichotomised at 65 years, sex, lactate dichotomised at 3.0 mmol/L, APACHE II score dichotomised at 15, source of infection (respiratory, urinary, other) and fluid volume prior to randomisation dichotomised at the median volume. A p value less than 0.05 (two-tailed) will be used to indicate statistical significance, including for the primary outcome. No adjustment for multiple tests will be performed. A cost-effectiveness analysis will be conducted, with a separate health economic analysis plan completed and published prior to data lock.

### Details of patient flow and characteristics, processes of care and clinical data

**Figure 2:**
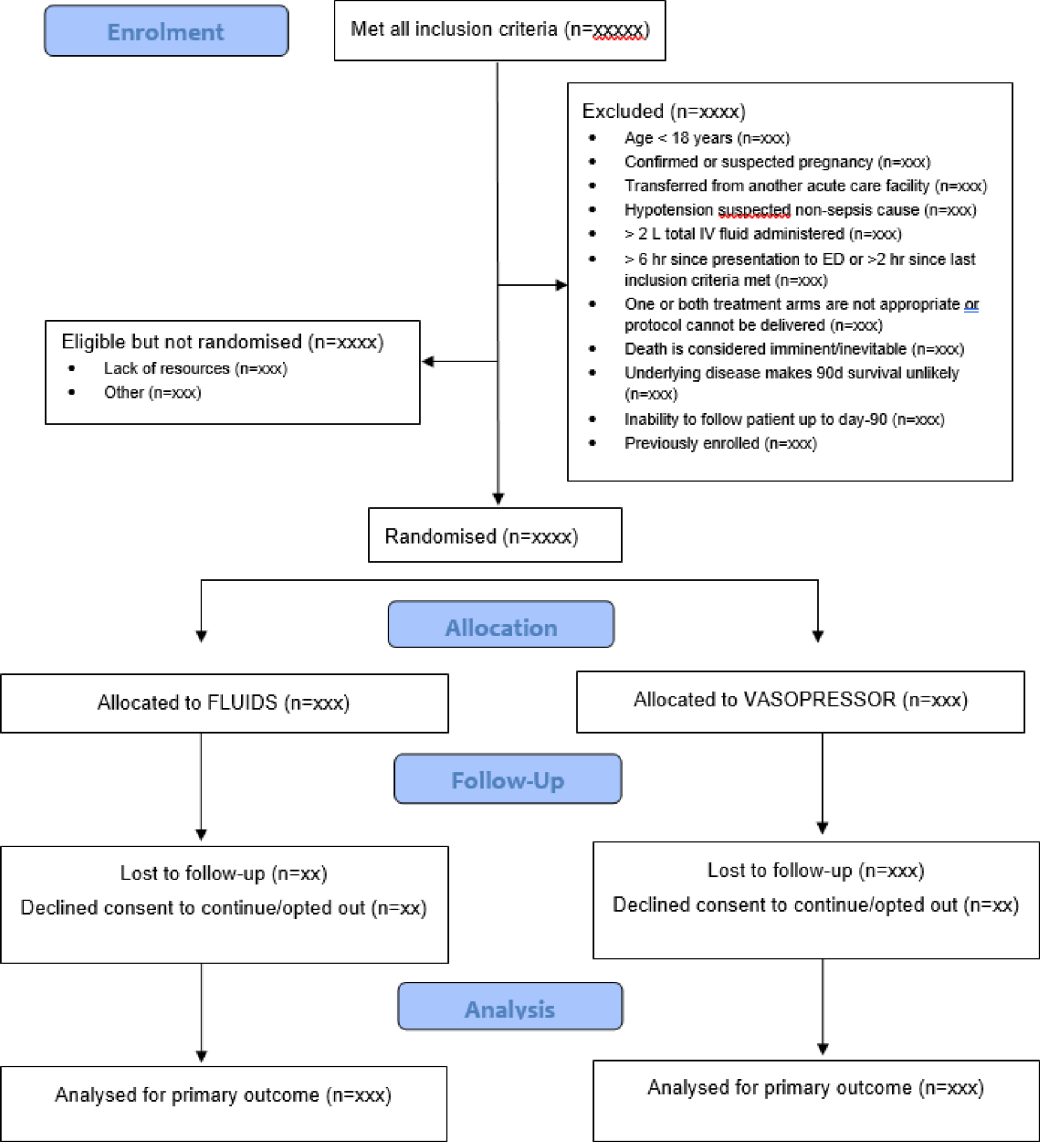
CONSORT diagram of participants in the ARISE FLUIDS trial. ARISE FLUIDS, Australasian Resuscitation In Sepsis Evaluation: FLUid or vasopressors In emergency Department Sepsis; CONSORT, Consolidated Standards of Reporting Trials; ED, emergency department; FLUIDS, fluids arm; VASOPRESSORS, vasopressor arm.

Flow through the trial will be presented in a Consolidated Standards Of Reporting Trials diagram (figure 2).^17^ We will report the numbers of patients who meet the trial eligibility criteria and the numbers excluded (including reasons for exclusion), randomised and available for evaluation of the primary outcome. Patient characteristics and details on study-specific interventions and physiologic and laboratory variables during the first 24 hours postrandomisation will be reported by treatment group. The following will also be reported by treatment group: microbiology (up to 72 hours post-ED admission) and ancillary treatments (eg, organ support, corticosteroid therapy), complications related to the study intervention (eg, central or peripheral venous catheter-related, ischaemia, acute pulmonary oedema) and adverse events up to hospital discharge.

## Ethics and dissemination

### Research ethics approval

Ethics approval was obtained prior to study commencement at each participating institution from the responsible local or national human research ethics committee. In Australia, it was approved by the Northern Sydney Local Health District Human Research Ethics Committee (2020/ETH02874) on 21 January 2021. In New Zealand, it was approved by Northern B Health and Disability Ethics Committee (21/NTB/40) on 3 May 2021 and in Ireland by St. Vincent’s Health Group Research Ethics Committee (RS21-055) on 22 September 2022.

### Consent

Approval was obtained for patients to be enrolled without prior informed consent on the basis that the two treatment options are acceptable as part of current standard care management for patients with septic shock. The patient or next-of-kin (or equivalent according to local jurisdiction) is approached at the first available opportunity and given a trial information sheet (online supplemental Appendix 6). According to local approvals, the patient or next-of-kin chooses to either continue in the trial or opt-out/decline continued participation. Where a decision is made to opt-out/decline continued participation, usual care is provided and no patient data are used, unless specific consent to do so is obtained. This approach accords with the Declaration of Helsinki for research involving time-critical interventions among people who do not have capacity to consent and is consistent with local ethics and regulatory requirements.

### Dissemination plan

The results will be disseminated by several media, including publications in peer-reviewed international medical journals, and presentations at national and/or international conferences.

## Supplementary material

10.1136/bmjopen-2025-101215 online supplemental file 1

10.1136/bmjopen-2025-101215 online supplemental file 2

10.1136/bmjopen-2025-101215 online supplemental file 3

10.1136/bmjopen-2025-101215 online supplemental file 4

10.1136/bmjopen-2025-101215 online supplemental file 5

10.1136/bmjopen-2025-101215 online supplemental file 6
