# Supplementary material for: Australasian Resuscitation In Sepsis Evaluation: FLUid or vasopressors In emergency Department Sepsis (ARISE FLUIDS) trial: study protocol
Source: BMJ Open. 2025 Jul 20;15(7):e101215. doi: 10.1136/bmjopen-2025-101215 (PMC12278162; doi:10.1136/bmjopen-2025-101215)
Supplement: online supplemental file 1 [file bmjopen-15-7-s001.pdf]

ARISE FLUIDS Management Committee	
**Member**	**Affiliation**
Prof Sandra L. Peake	Discipline of Acute Care Medicine, The University of Adelaide, Adelaide, South Australia, Australia;<br>Australian and New Zealand Intensive Care Research Centre, Monash University, Melbourne, Victoria, Australia;<br>Department of Intensive Care Medicine, The Queen Elizabeth Hospital, Woodville South, South Australia, Australia.
Clin/A Prof Stephen P. J. Macdonald	Centre for Clinical Research in Emergency Medicine, Harry Perkins Institute of Medical Research, Perth, Western Australia, Australia
A/Prof Glenn Arendts	Emergency Medicine, Fiona Stanley Hospital, Western Australia, Australia;<br>School of Medicine, University of Western Australia, Western Australia, Australia
Prof Rinaldo Bellomo	Intensive Care, Austin Health, Victoria, Australia
Mr Jonathan Burcham	Clinical Nurse Manager Emergency Research, Royal Perth Hospital, Western Australia, Australia
A/Prof Anthony Delaney	Intensive Care, Royal North Shore Hospital, Sydney, New South Wales, Australia;<br>Professorial Fellow, Critical Care Program, The George Institute for Global Health, University of New South Wales, New South Wales, Australia
Prof Diana Egerton-Warburton	Emergency Medicine, Monash Medical Centre, Melbourne, Victoria, Australia;<br>School of Clinical Science and Monash Health, Monash University Melbourne, Victoria, Australia
Prof Daniel Fatovich	Emergency Medicine, Royal Perth Hospital, Western Australia, Australia
Prof John Fraser	Critical Care Research Group, University of Queensland and The Prince Charles Hospital, Queensland, Australia
Dr Alisa M. Higgins	Senior Research Fellow, ANZIC-RC, Monash University, Melbourne, VIC, Australia
Ms Belinda D. Howe	Project Manager, ANZIC-RC, Monash University, Melbourne, Victoria, Australia
A/Prof Peter Jones	Emergency Medicine, Auckland Hospital, Auckland, New Zealand
Prof Gerben Keijzers	Department of Emergency Medicine, Gold Coast Hospital and Health Service, Gold Coast, Queensland, Australia;

	Faculty of Health Sciences and Medicine, Bond University, Queensland, Australia;<br>School of Medicine and Dentistry, Griffith University, Queensland, Australia
Dr Elissa Milford	Advanced Trainee Intensive Care, Sunshine Coast University Hospital, Queensland, Australia
Ms [redacted]	Consumer representative, Western Australia, Australia
Prof Andrew Udy	Intensive Care, The Alfred Hospital, Melbourne, Victoria, Australia
Ms [redacted]	Consumer representative, New South Wales, Australia
Ms Patricia Williams	Discipline of Acute Care Medicine, The University of Adelaide, Adelaide, South Australia, Australia;<br>Australian and New Zealand Intensive Care Research Centre, Monash University, Melbourne, Victoria, Australia;<br>Department of Intensive Care Medicine, The Queen Elizabeth Hospital, Woodville South, South Australia, Australia.
Dr Paul Young	Intensive Care, Wellington Hospital, Wellington, New Zealand
