# Supplementary material for: Australasian Resuscitation In Sepsis Evaluation: FLUid or vasopressors In emergency Department Sepsis (ARISE FLUIDS) trial: study protocol
Source: BMJ Open. 2025 Jul 20;15(7):e101215. doi: 10.1136/bmjopen-2025-101215 (PMC12278162; doi:10.1136/bmjopen-2025-101215)
Supplement: online supplemental file 3 [file bmjopen-15-7-s003.pdf]

ARISE FLUIDS

Australasian Resuscitation In Sepsis Evaluation: FLUid or vasopressors In emergency Department Sepsis

Protocol V3 Dated 10/10/2024

Clinical Trial Identifier: NCT04569942

Chief Investigators

Professor Sandra Peake
Director
Department of Intensive Care Medicine
The Queen Elizabeth Hospital
28 Woodville Road
Woodville, South Australia, 5011
AUSTRALIA
Phone: +61 8 82226463
Fax: +61 8 8222 6045
Email: sandra.peake@sa.gov.au

Clin A/Prof Stephen Macdonald
Clinical Research Fellow
Centre for Clinical Research in Emergency Medicine
Harry Perkins Institute of Medical Research
Royal Perth Hospital
Perth, Western Australia, 6000
AUSTRALIA
Phone: +61 8 9244 8458
Fax: +61 8 92241494
Email: stephen.macdonald@health.wa.gov.au

Coordinating Centre
The Australian & New Zealand Intensive Care Research Centre
Department of Epidemiology and Preventive Medicine
School of Public Health and Preventive Medicine, Monash University
Level 3, 553 St Kilda Road
Melbourne, Victoria, 3004
AUSTRALIA
Phone: +61 3 9903 0343
mailto:anzicrc@monash.edu.au

anzic research centre — Australasian College for Emergency Medicine — ANZICS Clinical Trials Group

THIS STUDY IS ENDORSED BY THE AUSTRALIAN AND NEW ZEALAND INTENSIVE CARE SOCIETY CLINCIAL TRIALS GROUP (ANZICS CTG) AND THE AUSTRALASIAN COLLEGE FOR EMERGENCY MEDICINE CLINCIAL TRIALS NETWORK (ACEM CTN)

TABLE OF CONTENTS

1. ABBREVIATIONS 5
2. SYNOPSIS 7
3. STUDY ADMINISTRATION STRUCTURE 9
3.1 Coordinating Centre and Data Management Centre 9
3.1.1 Responsibilities 9
3.2 Working Party 9
3.2.1 Responsibilities 9
3.2.2 Members 9
3.3 Management Committee 10
3.3.1 Responsibilities 10
3.3.2 Members 10
3.4 Statistician 11
3.5 International Collaborators 11
3.6 Contact Details 11
3.6.1 Coordinating centre 11
3.6.2 Project Manager 12
3.6.3 Chief investigators 12
3.7 Data Safety and Monitoring Committee 13
3.7.1 Members 13
3.5.2 Meetings 13
4. MANAGEMENT COMMITTEE AUTHORISATION PAGE 14
5. LAY DESCRIPTION 17
6. BACKGROUND AND RATIONALE 18
7. OBJECTIVES 24
7.1 Aim 24
7.2 Hypothesis 24
8. STUDY OUTCOME MEASURES 24
8.1 Primary outcome 24
8.2 Secondary outcomes 24
8.3 Tertiary outcomes 24
9. OVERALL STUDY DESIGN 25
9.1 Study design 25
9.2 Study population 25

9.3 Inclusion criteria 25
9.4 Exclusion criteria 26
9.5 Screening Log 26
9.6 Co-enrolment 26
10. STUDY PROCEDURES 26
10.1 Randomisation 26
10.2 Study Treatment arms 27
10.3 Vasopressors arm 27
10.4 Fluids arm 27
10.5 Safety and monitoring during intervention period for both study arms 28
10.5.1 Monitoring for fluid overload 28
10.5.2 Monitoring for hypovolaemia or hypoperfusion 28
10.6 Ancillary treatments 28
10.7 Disposition 29
10.8 Discontinuation of treatment 29
10.9 Follow up at day 90, 6 and 12 months 29
10.10 Bias Minimisation 29
11. ETHICS 30
11.1 Ethical conduct of the study 30
11.2 Ethical considerations of the study 30
11.3 The trial compares two standard care treatment arms 30
11.3.1 With regards to the NSW Guardianship Act. 31
11.4 Data protection and confidentiality of patient data 31
11.5 The enrolment of participants who are unable to consent for themselves 32
11.5.1 Urgent treatment for septic shock and trial inclusion unable to be separated 32
11.5.2 Unable to obtain patient or LAR consent prior to enrolment 32
11.5.3 Enrolment without prior consent 32
11.6 Informed consent procedures- Australian context 33
11.6.1 Opt-out approach 33
11.6.2 Consent to Continue 34
11.6.3 Deceased patients 34
11.6.4 Informed consent cannot be obtained from the participant or legally authorised representative 34
11.7 Informed consent procedures- New Zealand context 35

12. DATA MANAGEMENT 35
12.1 Data collection and management methods 35
12.2 Data variables collected 36
12.3. Monitoring 37
12.4 Protocol deviations 38
13. STATISTICAL CONSIDERATIONS 38
13.1 Sample size calculation 38
13.2 Statistical and analytical plan 38
13.3 Subgroup analyses 39
13.4 Interim analysis 39
14. SAFETY 39
14.1 Data Safety Monitoring Committee 39
14.2 Adverse events 39
14.3 Serious adverse events 40
14.4 Reporting 40
15. FUNDING 41
16. PUBLICATION POLICY 41
17. TRIAL REGISTRATION 41
18. REFERENCES 42
19. APPENDIX 1: Opt-Out approach and the national statement 45

1. ABBREVIATIONS

ACEM CTN	Australasian College for Emergency Medicine Clinical Trials Network
AE	Adverse event
ANP	Atrial Natriuretic Peptide
ANZIC-RC	Australian and New Zealand Intensive Care Research Centre
ANZICS CTG	Australian and New Zealand Intensive Care Society Clinical Trials Group
APACHE II	Acute Physiologic Assessment and Chronic Health Evaluation II
APO	Acute pulmonary oedema
ARISE FLUIDS	Australasian Resuscitation In Sepsis Evaluation: FLUid or vasopressors In emergency Department Sepsis
ARISE	Australasian Resuscitation In Sepsis Evaluation
CI	Confidence interval
CLASSIC	Conservative versus Liberal Approach to Fluid Therapy of Septic Shock in Intensive Care
CLOVERS	Crystalloid Liberal versus Vasopressors Early
CVC	Central venous catheter
DAOH-D90	Days alive and out of hospital to day 90
DSMC	Data Safety Monitoring Committee
E/CRF	Electronic/case report form
ED	Emergency department
EG	Endothelial glycocalyx
EQ-5D-5L	EuroQol- 5 Dimension- 5 Levels
HDU	High Dependency Unit
Hr	Hour
HREC	Human Research Ethics committee
ICH GCP	International Conference on Harmonization Good Clinical Practice
ICU	Intensive Care Unit
IPDMA	Individual patient data meta-analysis
IQR	Interquartile range
IV	Intravenous
Kg	Kilogram
MAP	Mean arterial pressure
ml	Millilitres
mmHg	Millimeters of mercury
mmol	Millimoles
NHMRC	National Health and Medical Research Council

L	Litres
LAR	Legally authorised representative
LPS	Lipopolysaccharide
QALY	Quality-adjusted life-year
REFRESH	Restricted Fluid Resuscitation in Sepsis associated Hypotension
RELIEF	Restrictive Liberal Fluid Therapy in Major Abdominal Surgery
SAE	Serious adverse events
SBP	Systolic blood pressure
SOFA	Sequential organ failure assessment score
SPHPM	School of Public Health and Preventive Medicine
SSC	Surviving Sepsis Campaign
USA	United States Of America
WHODAS	World Health Organization Disability Assessment Schedule

2. SYNOPSIS

Background	The optimal approach to haemodynamic resuscitation in patients with septic shock is uncertain. Conventional practice, supported by international consensus guidelines, is initial administration of 30 ml/kg of intravenous (IV) fluids for newly diagnosed septic shock. Emerging evidence of harm associated with excessive IV fluids has led to more recent variation in practice with some clinicians favouring smaller fluid volumes combined with early vasopressor administration to restore systemic blood pressure. The impact on clinical outcomes among patients with septic shock presenting to emergency departments in Australia and New Zealand is unknown.
Aim	To compare the effect of a strategy of restricted fluids and early introduction of vasopressors to a strategy which involves a larger initial volume of IV fluid and later vasopressor administration if required, for haemodynamic resuscitation of patients with early septic shock.
Objectives	To provide robust evidence for clinicians regarding the optimal approach to early haemodynamic resuscitation in septic shock to improve patient-centred clinical outcomes.
Methods	This multicentre, randomised controlled trial will enrol 1000 patients presenting with septic shock to the emergency department (ED) of participating hospitals in Australia and New Zealand. Participants will receive haemodynamic resuscitation with either a restricted fluids and early vasopressor regimen or a larger initial IV fluid volume with later introduction of vasopressors if required. Clinical care including the type of resuscitation fluid and vasopressor agent, will otherwise be in accordance with accepted standard care and according to clinician discretion. The study intervention will be delivered for at least 6 hours and up to 24 hours post-randomisation. Participants will be followed for up to 12 months and outcomes analysed on an intention-to-treat basis.
Outcomes	The **primary outcome** is days alive out of hospital at 90 days post-randomisation.<br>**Secondary outcomes** will be:<br>1. All-cause mortality at 90 days<br>2. Time from randomisation until death (to day 90)<br>3. Days alive and at home at 90 days post-randomisation<br>4. Ventilator-free days to day 28<br>5. Vasopressor-free days to day 28<br>6. Renal replacement therapy-free days to day 28<br>7. Death or disability at 6 and 12 months*

	**Tertiary outcomes** will include:<br>1. Incidence and duration of invasive mechanical ventilation<br>2. Incidence and duration of vasopressor support<br>3. Incidence and duration of acute renal replacement therapy<br>4. ED length of stay<br>5. ICU length of stay<br>6. Hospital length of stay<br>7. In hospital mortality (censored at 90 days)<br>8. Mortality at 6 and 12 months*<br>9. Quality of life at 6 and 12 months*<br>10. Cost-effectiveness measured as cost/QALY*<br>*May be reported separately when 12-month follow up complete
Trial Registration	ClinicalTrials.gov (NCT04569942)

3. STUDY ADMINISTRATION STRUCTURE

3.1 Coordinating Centre and Data Management Centre

Australian and New Zealand Intensive Care Research Centre (ANZIC-RC) and School of Public Health and Preventive Medicine (SPHPM), Monash University, Melbourne, Victoria, Australia.

3.1.1 Responsibilities

Responsible for all aspects of study management including:

- Assistance with human research ethics committee applications
- Management of study budget and liaison with funding bodies
- Protocol and case report form (CRF) design and production
- Database design and management
- Protocol training of investigators, research coordinators and the ARISE FLUIDS study team
- Preparation and arrangement of investigator payments
- Trial set-up
- Randomisation
- Coordination of data entry and feedback of data enquiries
- Monitoring and close-out visits
- Organisation of investigator meetings
- Serious adverse event notification
- Data analyses and collaboration on publications

3.2 Working Party

3.2.1 Responsibilities

Responsible for overseeing all aspects of study including:

- Liaison with coordinating centre and management committee
- Funding applications, negotiations and communications
- Budget reporting to funding bodies
- Approval of final protocol and data collection form
- General study management issues

3.2.2 Members

Chair

Clin/A Prof Stephen Macdonald   Royal Perth Hospital, Perth, WA

Investigators

Prof Sandra Peake	Department of Intensive Care Medicine, The Queen Elizabeth Hospital, Adelaide, SA
A/Prof Anthony Delaney	Intensive Care, Royal North Shore Hospital, Sydney, NSW
Dr Alisa Higgins	Research Fellow, ANZIC-RC, Monash University, Melbourne, VIC
Ms Belinda Howe	Project Manager, ANZIC-RC, Monash University, Melbourne, VIC
A/Prof Peter Jones	Emergency Medicine, Auckland Hospital, , NZ
Prof Gerben Keijzers	Emergency Medicine, Gold Coast University Hospital, Gold Coast, QLD
[redacted]	[redacted]
Prof Andrew Udy	Intensive Care, The Alfred Hospital, Melbourne, VIC
Ms Patricia Williams	Research Coordinator, Department of Intensive Care Medicine, The Queen Elizabeth Hospital, Adelaide, SA

3.3 Management Committee

3.3.1 Responsibilities

Responsible for overseeing all aspects of the study including:

- Liaison with coordinating centre staff and trial management staff
- Liaison with ANZICS CTG, ACEM CTN and Data Safety Monitoring Committee (DSMC)
- Overseeing funding applications
- Overseeing disbursement and administration of funds
- Ensuring fiscal responsibilities are maintained
- Development and approval of final protocol and trial materials
- Development and approval of data collection tools and methods
- Data analysis, reporting and approval of study publications

3.3.2 Members

Chair

Prof Sandra Peake	Department of Intensive Care Medicine, The Queen Elizabeth Hospital, Adelaide, SA

Investigators

Clin/A Prof Stephen Macdonald	Royal Perth Hospital, Perth, WA
A/Prof Glenn Arendts	Emergency Medicine, Fiona Stanley Hospital, WA
Prof Rinaldo Bellomo	Intensive Care, Austin Health, VIC
Mr Jonathan Burcham	Clinical Nurse Manager Emergency Research, Royal Perth Hospital, WA

A/Prof Anthony Delaney	Intensive Care, Royal North Shore Hospital, Sydney, NSW
Prof Diana Egerton-Warburton	Emergency Medicine, Monash Medical Centre, VIC
Prof Daniel Fatovich	Emergency Medicine, Royal Perth Hospital, WA
Prof John Fraser	Intensive Care, The Prince Charles Hospital, QLD
Dr Alisa Higgins	Research Fellow, ANZIC-RC, Monash University, Melbourne, VIC
Ms Belinda Howe	Project Manager, ANZIC-RC, Monash University, Melbourne, VIC
A/Prof Peter Jones	Emergency Medicine, Auckland Hospital, NZ
Prof Gerben Keijzers	Emergency Medicine, Gold Coast University Hospital, Gold Coast, QLD
[redacted]	[redacted]
Dr Elissa Milford	Advanced Trainee Intensive Care, Sunshine Coast University Hospital, QLD
Ms [redacted]	Consumer representative, WA
Prof Andrew Udy	Intensive Care, The Alfred Hospital, Melbourne, VIC
Ms [redacted]	Consumer representative, NSW
Ms Patricia Williams	Research Coordinator, Department of Intensive Care Medicine, The Queen Elizabeth Hospital, Adelaide, SA
Dr Paul Young	Intensive Care, Wellington Hospital, NZ

3.4 Statistician

Prof [redacted] ANZIC Research Centre, Monash University, Melbourne, VIC

The independent statistician is not a member of the management committee but may attend a meeting/s at the committee's request to provide statistical advice.

3.5 International Collaborators

Prof [redacted] [redacted]

Prof [redacted] [redacted]

3.6 Contact Details

3.6.1 Coordinating centre

The Australian and New Zealand Intensive Care Research Centre

School of Public Health and Preventive Medicine, Monash University
Level 3, 553 St Kilda Road
Melbourne, Victoria 3004

AUSTRALIA

Phone +61 3 9903 0343

Email anzicrc@monash.edu

Web http://anzicrc.monash.org

3.6.2 Project Manager

Ms Belinda Howe

The Australian and New Zealand Intensive Care Research Centre

Department of Epidemiology and Preventive Medicine

School of Public Health and Preventive Medicine, Monash University

Level 3, 553 St Kilda Road

Melbourne, Victoria 3004

AUSTRALIA

Phone +61 3 9903 0340

Email: Belinda.howe@monash.edu

3.6.3 Chief investigators

Professor Sandra Peake

Director, Department of Intensive Care Medicine

The Queen Elizabeth Hospital

28 Woodville Road

Woodville, South Australia, 5011

AUSTRALIA

Phone: +61 8 8222 6463

Fax: +61 8 8222 6045

Email: Sandra.Peake@sa.gov.au

Clin A/Prof Stephen Macdonald

Clinical Research Fellow, Centre for Clinical Research in Emergency Medicine

Harry Perkins Institute of Medical Research

Royal Perth Hospital

Perth, Western Australia, 6000

AUSTRALIA

Phone: +61 8 9244 8458

Fax: +61 8 9224 1494

Email: stephen.macdonald@health.wa.gov.au

3.7 Data Safety and Monitoring Committee

3.7.1 Members

DSMC Chair

[redacted]

DSMC Committee members

[redacted]

[redacted]

[redacted]

[redacted]

3.5.2 Meetings

DSMC meetings will be scheduled prior to trial commencement, and one pre-specified interim analysis scheduled following recruitment of 500 of the planned 1000 patients.

In addition, following the recruitment of the first 150 participants, a review will be undertaken of recruitment rates, protocol compliance and separation between groups for volume of IV fluids and timing of vasopressor commencement.

Other meetings will be scheduled by the DSMC as required at their absolute discretion.

4. MANAGEMENT COMMITTEE AUTHORISATION PAGE

We, the management committee, have read the attached protocol version 2.0, dated 15 DEC 2020 and authorise it as the official protocol for the study entitled "Australasian Resuscitation In Sepsis Evaluation: FLUid or vasopressors In emergency Department Sepsis".

Chair, Management Committee<br>Professor Sandra Peake	Date 10 OCT 2024
Management Committee<br>Clin/A Prof Stephen Macdonald	Date 10 OCT 2024
Management Committee<br>A/Prof Glenn Arendts	Date 10 OCT 2024
Management Committee<br>Prof Rinaldo Bellomo	Date 10 OCT 20204
Management Committee<br>Mr Jonathan Burcham	Date 10 OCT 2024
Management Committee<br>A/Prof Anthony Delaney	Date 10 OCT 2024
Management Committee<br>Prof Diana Egerton-Warburton	Date 10 OCT 2024
Management Committee<br>Prof Daniel Fatovich	Date 10 OCT 2024

Management Committee Date 10 OCT 2024

Prof John Fraser

Management Committee Date 10 OCT 2024

Dr Alisa Higgins

Management Committee Date 10 OCT 2024

Ms Belinda Howe

Management Committee Date 10 OCT 2024

A/Prof Peter Jones

Management Committee Date 10 OCT 2024

Prof Gerben Keijzers

Management Committee Date 10 OCT 2024

[redacted]

Management Committee Date 10 OCT 2024

Dr Elissa Milford

Management Committee Date 10 OCT 2024

Ms [redacted]

Management Committee Date 10 OCT 2024

Prof Andrew Udy

Management Committee Date 10 OCT 2024

Ms [redacted]

Management Committee<br>Ms Patricia Williams	Date 10 OCT 2024
Management Committee<br>Dr Paul Young	Date 10 OCT 2024

5. LAY DESCRIPTION

Sepsis is a life-threatening illness which arises when the body's response to an infection injures its own tissues and organs. Septic shock is a severe subset of sepsis characterised by low blood pressure such that the blood flow to tissues is inadequate. Septic shock is a medical emergency requiring urgent treatment to restore adequate blood flow to the tissues, as well as measures to control the underlying infection.

The conventional standard initial treatment for septic shock is to attempt to restore the blood pressure by giving IV fluids through a drip. International expert consensus guidelines recommend that 20-30 ml/kg (approximately 2-3 litres in an average adult) be administered during the first 3 hours of treatment. If this is not sufficient to correct the problem, an IV drip containing medication to raise the blood pressure is started. Many patients receive a combination of fluids and medications.

Despite the observation that there has been a reduction in deaths from sepsis in the past two decades, there is emerging evidence that IV fluids, particularly in excess quantities, are associated with harm in sepsis. An alternative is to give less fluid initially and start a medication drip earlier as a means of reducing the overall amount of fluid administered. There is currently uncertainty about which is best for patients, and as a consequence practice varies between these approaches. In addition, as mortality has fallen, there is an increasing focus on quality of life among survivors. People who survive septic shock can have severe lifelong problems including persistent organ failure, amputated limbs, psychological injury and impaired ability to function at the level they previously enjoyed.

This trial will investigate if giving a smaller amount of initial IV fluid with earlier commencement of a medication drip ("vasopressors") to improve blood pressure leads to better patient outcomes than the conventional approach of giving a larger amount of fluid and starting medication later ("fluids"). Patients eligible for the trial who have suspected septic shock in the ED will be randomly allocated to receive treatment according to one of these treatment regimens. We will follow patients up and assess their progress. The main outcome we will measure is the number of days the patient has survived out of hospital at 90 days after entering the trial. This outcome measure has been chosen in consultation with consumers as being a patient-centred measure which captures survival as well as being associated with severity of illness and quality of life.

The trial will recruit 1000 patients from emergency departments in metropolitan and regional hospitals across Australia and New Zealand.

6. BACKGROUND AND RATIONALE

Sepsis is a major public health problem

Identified as a Global Health Priority by the World Health Organisation [1], sepsis (defined as life-threatening organ dysfunction due to a dysregulated host reponse to infection) is estimated to affect 50 million people worldwide each year [2]. In industrialised countries, it accounts for significant mortality, morbidity, and healthcare expenditure [3]. The Australian Sepsis Network reports an annual incidence of sepsis requiring admission to an Intensive Care Unit (ICU) of 0.77/1000, approximately 18,000 cases, with an estimated cost per episode of $39,000 [4]. Data from the Australian and New Zealand Intensive Care Society (ANZICS) have shown that sepsis accounts for 11% of all ICU admissions, with an overall in-hospital mortality rate of 18%, and 22% in the subgroup with shock [5]. As mortality has fallen in the past two decades there is increasing awareness of the burden of morbidity among survivors [6]. One third of those treated in an ICU for sepsis have not returned to their previous level of function by six months [7].

Evolving understanding of sepsis pathogenesis: the role of fluid administration

The hallmark of sepsis, and the principal driver of morbidity and mortality, is organ failure resulting from a dysregulated host response to infection [8]. Septic shock is defined as a subset of sepsis in which particularly profound circulatory, cellular and metabolic abnormalities are associated with a greater risk of mortality than sepsis alone. In practice, septic shock is defined as a requirement for vasopressors to maintain a mean arterial blood pressure of 65 mmHg or greater and a serum lactate greater than 2 mmol/L, *despite adequate volume resuscitation*. Expert consensus guidelines from the Surviving Sepsis Campaign (SSC) recommend the administration of at least 30 ml/kg of IV fluid (2-3 L in a typical adult) for hypotension or lactate >4 mmol/L, with infusion of a vasopressor such as noradrenaline if the patient remains hypotensive during or following fluid resuscitation [9], however supporting evidence is limited and of low quality. Reductions in sepsis mortality over the past two decades have coincided with an increased emphasis on early, aggressive management including IV antimicrobials, liberal fluid administration and senior clinician review [10]. It is unknown to what extent these interventions are responsible for the reduced mortality, if this is related to improved sepsis recognition, or other factors such as changing definitions or a shifting spectrum of illness severity.

Is large volume IV fluid resuscitation harmful?

Data are emerging that the liberal use of IV fluids in sepsis may be associated with increased organ failure, ICU length of stay and mortality [11, 12]. A large randomised trial among children with a febrile illness and impaired perfusion in a resource-poor setting in Africa found early treatment with a 20-40 ml/kg bolus of IV fluid (0.9% saline or 5% albumin) resulted in increased mortality compared to children that received only maintenance fluid with no bolus [13]; 10.5% *v* 7.3% at 48 hours, odds ratio (OR) 1.45, 95% CI 1.13 to 1.86, p=0.003. Although a fluid bolus improved clinical perfusion parameters in the short term, the excess of deaths was attributed to cardiovascular collapse [14].

In 2017, Andrews *et al.* published the results of a randomised trial of a protocolised approach to resuscitation, including mandated fluid boluses among adult septic shock patients presenting to the ED of a Zambian hospital (n=212) [15]. The mortality rate in the protocolised care group, who received a median fluid volume in the first 6 hours of 3.5 L (Interquartile range [IQR] 2.7-4.0 L) was higher than in the usual care group who received a median of 2.0 L (IQR 1.0-2.5 L), 48% v 33%, relative risk 1.46 (95% CI 1.04-2.05, p=0.03 (Figure 1).

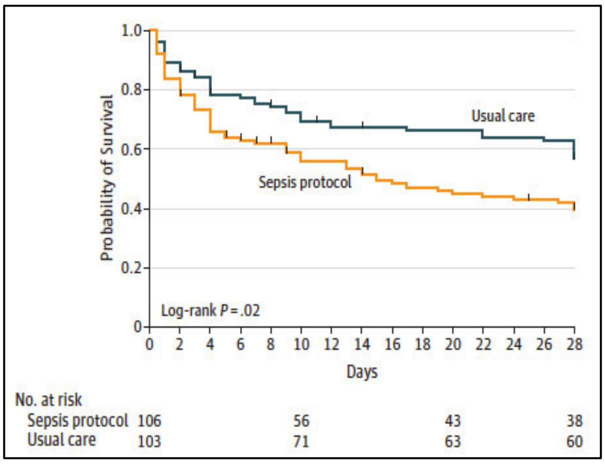

Figure 1. Kaplan-Meier curves for survival for protocolised sepsis resuscitation (median 3.5 L over 6h) compared to usual care (median 2.0 L over 6h) among Zambian ED patients with septic shock [15].

Mechanistic plausibility: role of the glycocalyx and endothelium

Fluid boluses may be harmful in sepsis by flushing mediators out of closed capillary beds, exacerbating the inflammatory insult. IV fluid also affects the structure and function of endothelial cells within the walls of small blood vessels [16]. Endothelial cell activation has been linked with adverse outcomes in sepsis [17]. A critical step is loss of the protective endothelial glycocalyx (EG) coating on the luminal surface [18]. Degradation of this mesh-like structure of proteoglycans and glycosaminoglycans increases interaction between leucocytes, platelets and the endothelium, increasing vascular permeability and propagating systemic inflammation in sepsis. Glycocalyx degradation has been postulated as a consequence of a fluid bolus, both directly and mediated by Atrial Natriuretic Peptide (ANP) released in response to cardiac stretch [19]. This raises the possibility that fluid boluses may contribute to inflammation and organ dysfunction.

Pre-clinical evidence that fluid resuscitation may be harmful in septic shock

A pre-clinical trial induced experimental septic shock by IV injection of lipopolysaccharide (LPS) in an ovine model, with subsequent randomisation to fluid resuscitation with normal saline (40 ml/kg), or no fluid resuscitation. This demonstrated that an initial fluid bolus is associated with (a) myocardial damage with raised troponin levels; (b) EG damage and raised ANP levels; and (c) increased vasopressor requirements [20]. These

findings challenge the current paradigm and are consistent with the results of the two African trials. This further supports the concept that fluid boluses are not only ineffective for treating hypotension, but may actually exacerbate shock in the setting of sepsis.

Support for a volume-sparing/early vasopressor approach

Accumulating evidence from observational studies, experimental models, and randomised trials in resource-poor countries challenge the standard approach to early fluid volume resuscitation recommended by the SSC. While there is currently no high-quality evidence to guide practice in the ED in industrialised countries, the question has been addressed in ICU populations. In a meta-analysis of 11 clinical trials, Silversides *et al* found a fluid restrictive approach in ICU was associated with increased ventilator-free days and reduced ICU length of stay [21].

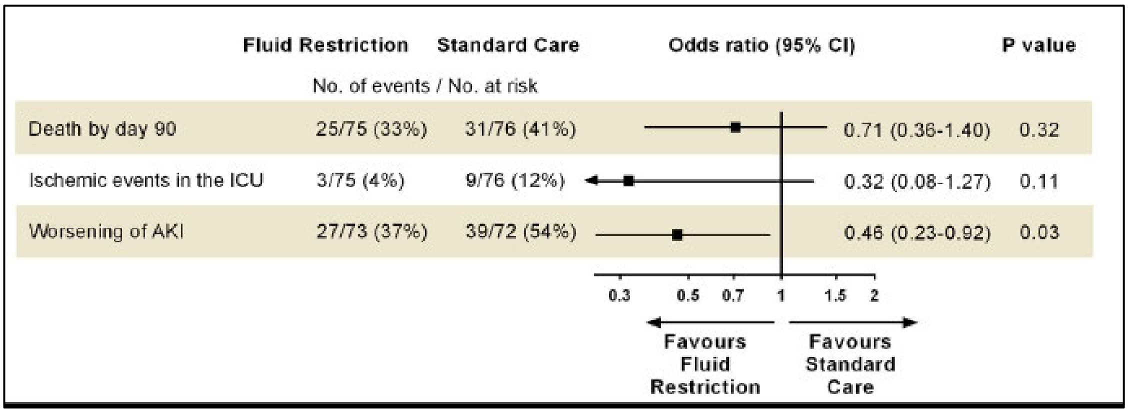

Figure 2. Clinical outcomes among 151 ICU patients randomised to either fluid restriction or usual care fluid resuscitation for septic shock in the CLASSIC trial; median (IQR) fluid volume in first 5 days post-randomisation, 0.5(0-2.5) L vs 2.0(1.0-4.1) L [22].

Hjortrup *et al* published a pilot clinical trial of restricted fluid boluses versus usual care among 151 ICU patients with septic shock in Denmark and Finland (Figure 2) [22]. A lower resuscitation fluid volume administered during the ICU stay coincided with a significant reduction in renal injury and non-significant reductions in ischaemic events and mortality. Over 4 L of fluid was administered prior to randomisation. While none of these trials can inform resuscitation practices in the ED, they have prompted critical re-evaluation of the role of fluids in septic shock resuscitation globally.

A recently completed systematic review and meta-analysis of 621 patients with sepsis randomised to a fluid restricted versus usual care fluid regimen in 9 clinical trials between 2015 and 2018 was conducted[23]. The pooled OR for mortality showed no difference between the two regimens but the treatment effect estimate favoured fluid restriction (0.87 [95% CI 0.69, 1.10]).

Li *et al* published a systematic review and meta-analysis of studies comparing early versus later inititation of vasopressors in patients with septic shock [24]. In 5 studies involving 929 patients the early vasopressor strategy was associated with a lower volume of fluid in the first 6 hours, a shorter time to achieve target MAP and lower short term mortality. There was however substantial hereogeneity, and only 2 of the 5 studies were clinical trials.

Is a liberal IV fluid approach of benefit? Lessons from perioperative medicine

The multicentre Restrictive Liberal Fluid Therapy in Major Abdominal Surgery (RELIEF) trial randomised 3000 high-risk patients (the majority from Australia and New Zealand) undergoing elective major abdominal surgery to a liberal *versus* restricted perioperative fluid management regimen [25]. Although there was no difference in the primary outcome of 1-year disability-free survival, contrary to expectations based on previous smaller trials, the rates of surgical site infections and acute kidney injury (including need for dialysis) were higher in the restricted fluid group. Translation to patients with septic shock in the ED is not possible. Nevertheless, this demonstrates the importance of undertaking high-quality, large, randomised, multicentre clinical trials where equipoise exists [26].

Relationship of ARISE FLUIDS with current international trials

The Crystalloid Liberal versus Vasopressors Early (CLOVERS) trial is currently addressing the effect of these two resuscitation strategies on mortality among ED patients with suspected sepsis in the USA https://clinicaltrials.gov/ct2/show/NCT03434028. Resuscitation practices in the USA typically involve a larger initial fluid volume compared to Australia and New Zealand [27]. We are also interested in the effect on the *quality of survival* and have chosen outcome measures appropriate for this objective. Thus, differences in population characteristics, usual resuscitation practice and outcomes of interest necessitate a stand-alone trial.

The European *Conservative versus Liberal Approach to Fluid Therapy of Septic Shock in Intensive Care (CLASSIC)* trial https://clinicaltrials.gov/ct2/show/NCT03668236 is assessing the effect of a fluid restricted resuscitation regimen upon 90-day mortality among patients admitted to the ICU following initial resuscitation. The trial does not therefore address the question of the optimal approach to initial resuscitation.

The principal investigators of the CLOVERS and CLASSIC trials are protocol advisors to the ARISE FLUIDS trial. We aim to harmonise relevant data points and outcome measures to facilitate a future individual patient data meta-analysis (IPDMA) akin to that which was undertaken for the trilogy of early goal-directed therapy trials [28].

Preliminary data and feasibility

We recently completed an observational study of initial sepsis resuscitation practices in 70 hospitals in Australia and New Zealand (n=591) [29]. Among those with septic shock (n=82), in-hospital mortality was 8% (95% CI 3-

15%). A median volume of 4.8 L (IQR 3.9-6.2 L) was given in the first 24 hours. The fluid volume prior to commencing vasopressors was 2 L (IQR 1.2-3.0 L). Even accounting for confounders such as source of infection, age and comorbidity, this study demonstrates considerable variation in usual care and scope to undertake a trial comparing a smaller initial volume of fluid combined with earlier vasopressor use to a larger initial volume and later vasopressor administration to restore systemic arterial blood pressure in patients with early septic shock.

A vasopressor infusion conventionally requires insertion of a central venous catheter (CVC). In the ARISE trial, 40% of patients initially commenced vasopressors via a peripheral IV catheter prior to CVC insertion [30], particularly for sicker patients with shock [31]. We performed a systematic review which verified the safety of this approach [32]. Accordingly, initial peripheral vasopressor administration in our trial will facilitate earlier introduction and avoid unnecessary CVC insertion where vasopressors are required for only a short period of time.

Finally, we have completed a multicentre, pilot, randomised, clinical trial in patients presenting to ED with sepsis-induced hypotension; the Restricted Fluid Resuscitation in Sepsis associated Hypotension (REFRESH) trial [33]. Patients with sepsis and hypotension persisting after 1000 mls IV fluid, were randomised to a further fluid bolus (standard volume) or to restricted fluids plus vasopressors if required (n=94). The mean fluid volume administered pre-randomisation in the restricted and standard arms was 1.3±0.3 L and 1.4±0.4 L respectively. The mean total volume in the first 6 hours (including pre-randomisation) was 2.3±0.9 L (31±13 ml/kg) in the restricted arm versus 3.1±1.1 L (46±20 ml/kg) in the standard volume arm, p=0.0001. Rates of vasopressor use were similar, but median duration of vasopressor support was 20 (IQR 9-43) hours and 33 (IQR 13-50) hours respectively. With the appropriate personnel and financial support for the ARISE FLUIDS trial we expect to be able to reliably deliver the intervention and achieve clinically important separation between the comparator groups in the acute care setting.

Validity of trial primary outcome

Septic shock is associated with significant mortality and reduced quality of life in survivors. The primary outcome of days alive and out of hospital to day 90 (DAOH-D90) is a patient-centred outcome measure that will capture both death and morbidity. DAOH has been validated in other settings where mortality alone is insufficient to capture the burden of disease, particularly the longer term functional outcome sequelae [34, 35]. The choice of DAOH-D90 was informed by extensive consumer consultation with time spent out of hospital and at home considered to be an important outcome. DAOH is readily quantifiable and is currently being used as an outcome measure in another critical care trial [36]. A between group difference of 7 days in DAOH-D90 was informed by data from the ARISE randomised clinical trial and the ARISE FLUIDS observational study and is considered to be biologically plausible, clinically meaningful and important to sepsis survivors. In addition to capturing the overall burden of disease both in hospital and after discharge, a 7-day reduction in DAOH-D90 will have significant resource and cost implications.

Significance

On the basis of the existing evidence, there is equipoise about the effect of a fluid-restricted and early vasopressor strategy compared to a more liberal fluids and later introduction of vasopressors strategy on sepsis mortality and on longer term functional outcome among survivors. In addition, the organisational and resource implications for acute health systems of these approaches are unknown. The ARISE FLUIDS trial will address an important gap in the evidence base regarding the early haemodynamic management of patients with septic shock, provide clear guidance for bedside clinicians and optimise important patient-centred outcomes.

If it can be demonstrated that a volume-sparing approach increases the number of days-alive out of hospital, the potential impact on global resource use in the healthcare system will be substantial. We will undertake a detailed economic evaluation and follow patients up to assess their functional outcome at 12 months, a time frame which is relevant for consumers and supported by literature. The collaboration between researchers in the ANZICS CTG and the ACEM CTN will be further strengthened. Harmonisation with collaborative trials overseas will place Australia and New Zealand in the vanguard of a worldwide research effort to reduce the human and economic burdens of this World Health Organisation Global Health Priority disease.

7. OBJECTIVES

7.1 Aim

The primary aim of the study is to compare the effect of a strategy of restricted IV fluid volume and earlier introduction of vasopressors (vasopressors) to a strategy which involves a larger initial IV fluid volume and later introduction of vasopressors if required (fluids) to restore systemic arterial blood pressure, for haemodynamic resuscitation of patients with early septic shock presenting to the ED in Australian and New Zealand hospitals.

7.2 Hypothesis

We hypothesise that a restricted intravenous fluid volume and earlier introduction of vasopressors approach, compared to a larger initial intravenous fluid volume and later introduction of vasopressors approach to restore systemic arterial blood pressure, will increase the number of days alive and out of hospital at day 90 in patients presenting to the ED with early septic shock.

8. STUDY OUTCOME MEASURES

8.1 Primary outcome

The primary study outcome is the number of days alive out of hospital at 90 days post randomisation (DAOH-D 90). Participants who die on or prior to day 90 will be assigned zero days alive and out of hospital.

8.2 Secondary outcomes

1. All-cause mortality at 90 days
2. Time from randomisation until death (to day 90)
3. Days alive and at home at 90 days post-randomisation
4. Ventilator-free days to day 28
5. Vasopressor-free days to day 28
6. Renal replacement therapy-free days to day 28
7. Death or disability at 6 and 12 months*

8.3 Tertiary outcomes

1. Incidence and duration of invasive mechanical ventilation
2. Incidence and duration of vasopressor support
3. Incidence and duration of acute renal replacement therapy
4. ED length of stay
5. ICU length of stay
6. Hospital length of stay
7. In hospital mortality (censored at 90 days)

8. Mortality at 6 and 12 months*
9. Quality of life at 6 and 12 months*
10. Cost-effectiveness measured as cost/QALY*

*May be reported separately when 12-month follow up complete

9. OVERALL STUDY DESIGN

9.1 Study design

The ARISE FLUIDS study is a multicentre, randomised, parallel group clinical trial of a restricted fluids and early vasopressor strategy compared to a larger initial IV fluid volume and later vasopressors for the haemodynamic resuscitation of patients with septic shock presenting to the ED. It will be conducted in hospitals in Australia and New Zealand with 1000 patients recruited over a 3-year period.

Each patient meeting all of the inclusion and none of the exclusion criteria will be randomised to receive haemodynamic resuscitation using either a restricted fluid and early vasopressor regimen (vasopressors arm) or a larger initial fluid resuscitation volume (fluids arm) followed by later introduction of vasopressors (if required). The intervention will be commenced in the ED and delivered for at least 6 hours, and up to 24 hours post-randomisation if admitted to the ICU or other critical care area where the study protocol can be faithfully delivered. Treatment will revert to usual care as determined by the treating clinician when the patient is transferred to a non-critical care ward. All enrolled participants will be followed up and assessed for the defined study outcomes.

Participants will be identified using a systematic approach to screening and assessment of patients with possible sepsis presenting to the ED in accordance with standard clinical practice.

9.2 Study population

Adult patients admitted to the ED of a participating institution with septic shock. Eligible patients must meet the inclusion criteria within the first 6 hours of ED presentation and must be randomised within 2 hours of meeting the last inclusion criterion.

9.3 Inclusion criteria

Patients will be eligible if ALL of the following criteria are met while in the ED:

1. Clinically suspected infection
2. Systolic blood pressure (SBP) <90 mm Hg <u>or</u> mean arterial pressure (MAP) <65 mm Hg, despite a ≥1000ml cumulative total bolus of IV fluid administered over a maximum of 60 minutes; including pre-hospital boluses
3. Arterial or venous blood lactate >2.0 mmol/L
4. At least one dose of an IV antimicrobial has been commenced

9.4 Exclusion criteria

Patients will be excluded if ANY of the following apply:

1. Age < 18 years
2. Confirmed or suspected pregnancy
3. Transferred from another acute care facility
4. Hypotension suspected to be due to a non-sepsis cause
5. >2L total IV fluid administered (including prehospital fluids but excluding drugs and flushes)
6. More than 6 hours has elapsed since presentation to the ED or more than 2 hours has elapsed since last inclusion criterion has been met
7. Treating clinician considers that one or both of the treatment regimens are not suitable for the patient or the study protocol cannot be delivered e.g. limitation of care, requirement for immediate surgery
8. Death is considered imminent or inevitable
9. Underlying disease that makes survival to 90 days unlikely
10. Inability to follow patient up to day-90 e.g. unstable accommodation, overseas visitor
11. Previously enrolled in this study

9.5 Screening Log

All patients who meet all the inclusion criteria will be entered into the screening log. Those who are excluded will have all of their reason/s for exclusion recorded.

9.6 Co-enrolment

Co-enrolment in another intervention trial evaluating haemodynamic resuscitation for septic shock is not permitted.

Co-enrolment in other interventional studies will be considered by the Management Committee as required.

10.STUDY PROCEDURES

10.1 Randomisation

Randomisation will be performed by the site investigator, research coordinator or delegated staff at each site. This will occur through a dedicated, secure, password-protected, encrypted website which is available 24 hours, 7 days a week. Patients will be randomised using computer-generated, block randomisation sequence with variable block sizes and stratified by participating centre. Patients will be assigned in a 1:1 ratio to either: the Vasopressors arm or Fluids arm. Upon randomisation, the website will assign a unique patient study number.

10.2 Study Treatment arms

All participants will have received a minimum of 1 L within 1 hour but not more than 2L of resuscitation fluids (including pre-hospital fluids) prior to randomisation. The study allocated treatment arm, either Vasopressors or Fluids, will run for at least 6 hours and up to a maximum of 24 hours post-randomisation whilst present in a critical care area (ED, ICU, HDU). After this time or discharge to a general ward area, whichever occurs first, care will revert to standard management as determined by the treating clinician. Choice of resuscitation fluids is at treating clinician discretion according to local practice.

Patient safety is paramount and at all times the precise timing and resuscitation targets will be at the discretion of the treating team.

10.3 Vasopressors arm

A restricted fluids and early vasopressor strategy:

Cease IV fluid resuscitation. If persisting hypotension and/or hypoperfusion **commence a vasopressor infusion** (e.g. noradrenaline) and titrate according to local practice to achieve target MAP. The target MAP will be determined by the treating clinician. Reassess at least hourly for up to 6 hours post-randomisation, then as clinically required in conjunction with the protocol. Boluses of **250ml** of IV fluids are permitted if deemed indicated by the treating clinician, including for the following reasons:

- Refractory hypotension
- Persistent hypoperfusion e.g. delayed capillary refill time
- Lactate >4 mmol/L and/or rising from previous level despite at least 2 hours of resuscitation
- Persistent tachycardia
- Oliguria <0.5 ml/kg/hr for at least 2 hours.

Maintenance fluids are strongly discouraged.

10.4 Fluids arm

A larger initial intravenous fluid volume and later introduction of vasopressor strategy:

An fluid bolus of up to 1000ml will be administered over a maximum of 1 hour, if required, for persisting hypotension and/or hypoperfusion. Reassess at least hourly to 6 hours post-randomisation, then as clinically required in conjunction with the protocol. Further IV fluid boluses of **500ml** are recommended as clinically indicated to achieve the target MAP. The target MAP will be determined by the treating clinician. Haemodynamic resuscitation will be guided by usual clinical assessment including vital signs, mentation, perfusion, and urine output until the treating clinician determines fluid resuscitation is no longer clinically required. A **minimum of**

2-3 L (30 ml/kg), including pre-randomisation fluids, is recommended within 3 hours of ED arrival consistent with the SSC guidelines, unless clinically contraindicated [8]. Vasopressors may be commenced if blood pressure remains below target *despit*e optimal fluid resuscitation as determined by the treating clinician.

If the patient is *in extremis,* vasopressors may be commenced concurrently with fluids. Maintenance fluids may be administered if indicated, in accordance with local practice.

10.5 Safety and monitoring during intervention period for both study arms

10.5.1 Monitoring for fluid overload

Participants in both arms of the study will be **monitored for signs of fluid overload,** and fluid administration ceased or reduced based upon clinical assessment by the treating clinician. Signs of fluid overload include peripheral oedema, increased bilateral inspiratory auscultatory crackles, and radiographic evidence of pulmonary oedema.

10.5.2 Monitoring for hypovolaemia or hypoperfusion

Participants in both study arms will be **monitored for signs of hypovolaemia or hypoperfusion** and managed with additional IV fluids in accordance with the trial protocol. If a participant develops evidence of central (e.g. bowel) or peripheral (limb or digit) ischaemia or mottling, treat accordingly and continue the protocol where clinically appropriate. Signs of hypovolaemia or hypoperfusion may include persistent tachycardia, altered mentation**,** reduced capillary refill time and oliguria.

10.6 Ancillary treatments

All patients will receive additional treatment for sepsis according to clinician discretion and the SSC guidelines including airway and ventilator management, early broad-spectrum antimicrobial agents and source control where required. All patients will be managed initially in a critical care ward (ED, ICU, HDU) until their condition stabilises. The type of fluid administered and the use of therapies such as inotropic agents or corticosteroids will be at senior treating clinician discretion. To avoid unnecessary placement of a CVC, peripheral IV administration of vasopressors for up to 24 hours in accordance with local policies and guidelines is permitted.

Physiological and laboratory data will be collected where this is available as part of usual care. No additional blood tests or procedures are required for study purposes.

10.7 Disposition

We expect that the majority of participants in the trial will be admitted to ICU or HDU from the ED. In keeping with the pragmatic nature of the trial, the ultimate decision about admission disposition will be at the discretion of the treating clinicians.

10.8 Discontinuation of treatment

The trial intervention arms will be continued for at least the first six hours post-randomisation. For those admitted to ICU the interventions will continue up to 24 hours post-randomisation, or until transfer to a general ward, whichever occurs first. Following cessation of the study intervention, haemodynamic management will be according to usual practice.

10.9 Follow up at day 90, 6 and 12 months

The descriptor for the person who is legally allowed to give consent will vary between jurisdictions. For the purposes of this protocol, the descriptor 'legally authorised representative' (LAR) describes the person who is legally allowed to give consent for the participant.

The participant or a LAR, if the participant has not regained capacity, will be contacted via a telephone call at 90 days, 6 and 12 months after randomisation for study follow up.

The day 90 follow up will be to establish survival at this time point and additionally to collect important information on readmissions and discharge to other hsospitals, rehabilitation hospitals or long term nursing care facilities. This will inform the primary outcome for the study. As this information will be collected from the participant or (LAR), a diary/information collection sheet will be provided prior to hospital discharge to assist with this information collection.

At 6 and 12 months after randomisation, the participant or LAR will be contacted again via a phone call. This call will collect information quality of life and functional outcome information by the E-Q-5D and WHODAS 2.0 surveys.

10.10 Bias Minimisation

Given the nature of the intervention, it is impossible to blind treating clinicians and participants for logistical and safety reasons. Study treatment assignment will be revealed to treating staff as they are required to apply their clinical judgement in the delivery of the trial protocol.

Bias will be minimised by:

1. ensuring concealment of the treatment allocation until completion of the randomisation procedure
2. regular monitoring and reporting of protocol compliance and

3. an objective primary outcome measure which is not susceptible to ascertainment bias.

11.ETHICS

11.1 Ethical conduct of the study

This study is to be performed in accordance with the ethical principles of the Declaration of Helsinki (June 1964 and amended 1975, 1983, 1989, 1996, 2000, 2008 and Note of Clarification 2002 and 2004), ICH GCP Notes for Guidance on Good Clinical Practice (CPMP/ICH/135/95) E6(R2) annotated with Therapeutic Goods Administration comments, NHMRC National Statement on Ethical Conduct in Research Involving Humans 2007 (Updated 2018), the New Zealand Interim Good Clinical Research Practice Guidelines (Volume 2 1998 and Volume 3 2000).

Ethics approval will be obtained from all relevant ethics committees in each jurisdiction/country that is participating. Each participating site will submit the protocol and other relevant study documentation to the responsible local governance office for site-specific assessment. Approval of the protocol and related documents will be obtained prior to the start of the study. It is the principal investigators responsibility to ensure that all conditions for approval of the study are met and that amendments to the protocol or serious adverse events are also reported to the HREC as required by that committee. The inclusion in the trial of adults with incapacity to consent will be governed in accordance with the legal jurisdiction of each participating site.

11.2 Ethical considerations of the study

The ethical considerations of this study are:

- The risk/benefit ratio of the study treatment.
- Data protection and confidentiality of participant data.
- The enrolment of participants who are unable to consent for themselves

11.3 The trial compares two standard care treatment arms

Our observational study demonstrated variation in haemodynamic resuscitation practice among participants with septic shock among Australian and New Zealand EDs ranging between a fluid restricted and early vasopressor strategy and a liberal fluid and later vasopressor approach. The resuscitation regimens in the two arms of this trial *encompass this range of usual care*, and this trial is therefore comparing the effectiveness of these two strategies accepted by substantial numbers of medical practitioners specialising in the area of practice concerned. Participants in both arms of the trial will therefore be receiving treatment within the bounds of accepted standard clinical practice with no components of care being new or experimental. The treating clinician is expected to have equipoise about which of the two treatment strategies to employ in order to enrol a participant into the trial. A clinical contraindication to employing either or both of the treatment strategies is an explicit exclusion criterion. Regardless of the treatment arm, and, because the trial is unblinded, the treating doctor retains ultimate control over the volume of resuscitation fluid and the timing of vasopressors within the

parameters recommended in the study protocol. The treating doctor may deviate from the treatment protocol if deemed to be in the interest of the participant (with the reasons for this documented in the case report form). Thus, participants in the trial are considered to be at no greater risk than if they were receiving treatment outside the trial. Thus the risk/benefit ratio of the trial where both trial arms are encompassed by the range of usual care and is accepted as such by medical practitioners specialising in the area of emergency medicine and intensive care in Australia and New Zealand confers no greater clinical risk than receiving routine treatment.

11.3.1 With regards to the NSW Guardianship Act.

Acknowledging advice from the Civil and Adminstrative Tribunal of NSW in the case of Shehabi v Attorney General (NSW) [2016] NSWCATAP 137 [37], we note that the tribunal defines a clinical trial for the purposes of the Guardianship Act 1987 (NSW), s 33(1) as "a trial of drugs or techniques that necessarily involves the carrying out of new medical or dental treatment that has not yet gained the support of a substantial number of medical practitioners or dentists specialising in the area of practice concerned." The ARISE-FLUIDS trial will compare two resuscitation strategies that use intravenous fluids and vasopressors for the resuscitation of patients with early septic shock. Intravenous fluids and vasopressors used are well established treatments for patients with early septic shock [38,39]. These treatments are supported by the majority of practitioners in the field, as evidenced by observational studies,[38,39] as well as being recommended by the NSW Clinical Excellence Commission Sepsis Pathway [40]. Given that the treatments administered in the ARISE-FLUIDS trial are not new treatments and they are widely accepted by the majority of practitioners specialising in the area of practise concerned, we submit that the ARISE FLUIDS trial does not meet the established criteria to be considered a clinical trial according to the NSW Guardianship Act 1987 (NSW), s 33(1).

11.4 Data protection and confidentiality of patient data

All investigators and research staff will comply with the legislative requirements of their jurisdiction with regard to the collection, storage, processing and disclosure of personal information. Confidentiality of all participant data will be maintained by the use of unique identifiers, password protected electronic databases, secure storage of records and precautions to control access to authorised personnel only. All records will be kept in compliance with local ethical and research governance policies.

Participants will be randomised via a secure database and allocated a unique study number. The site research coordinator will compile an enrolment log which contains identifying information. Subsequent data will be identified by the unique study number only. The enrolment log and study data will be kept separately. Follow-up details of the participant and their LAR (if the participant has not regained adequate capacity) will be collected for the 6- and 12- month outcome assessment conducted via telephone including name, address and contact telephone numbers/email. These contact details will be kept confidentially at the site in a locked filing cabinet in the research office and will not be accessible outside the research team of the recruiting hospital.

11.5 The enrolment of participants who are unable to consent for themselves

Who may give consent for a patient to take part in medical research will vary between legal jurisdictions according to their local legislation. Each site's Principal Investigator is responsible for ensuring that the relevant local legal requirments are followed. The term used to describe the person who may give consent for a patient to take part in medical research will also vary between jurisdictions. For the purposes of this protocol, the descriptor 'legally authorised representative' describes the person who is legally allowed to give consent for the participant.

All interaction between research staff and participants and their relatives will take into consideration the stress or emotional factors associated with critical illness and ensure that the dependency of potential participants and their relatives on medical personnel providing treatment does not compromise the freedom of a decision to participate. Consenting to participation will be voluntary and participants or their legally authorised representative (LAR) will be free to withdraw from participation at any time without giving reasons.

11.5.1 Urgent treatment for septic shock and trial inclusion unable to be separated

Due to the critical nature of septic shock, treatment needs to be commenced rapidly under emergency conditions and is part of life-saving care. Additionally, the study treatment arms, both which encompass standard care for septic shock, cannot be separated from life saving care.

11.5.2 Unable to obtain patient or LAR consent prior to enrolment

The patient with septic shock is critically ill, requires time critical care and due to their condition is unable to provide valid and reliable consent. The patient's legally authorised representative (LAR) is often not present at this time or in many cases is not known or contactable. If, however, the LAR were present or contactable, taking into consideration the stress and emotional factors involved it would not be practical, nor feasible to obtain an informed consent in a respectful manner. The burden of this situation would be unfair to the LAR as detailed in the National Statement on Ethical Conduct in Human Research 2007 (updated 2018) section 4.4.11 [41].

11.5.3 Enrolment without prior consent

In view of the above, we will seek approval for enrolment without prior consent where this is consistent with local jurisdictional requirements and legislation. This approach is in line with the principles in paragraph 4.4.13 of the National Statement on Ethical Conduct in Human Research and is justified on the basis that the trial is *comparing the effectiveness of two accepted treatment strategies and trial participation confers no greater clinical risk than receiving routine treatment, as detailed in protocol section 11.3.*

Enrolment will only occur provided the following conditions are met:

- The patient's condition requires urgent treatment which cannot be delayed
- The patient meets all trial eligibility criteria with no exclusions

- It is not possible to obtain an informed, respectful consent from the legally authorised representative without delaying treatment
- The research project is not contrary to the interests of the patient.

These above conditions will be documented in the medical record.

11.6 Informed consent procedures- Australian context

As soon as reasonably practicable following recruitment, the LAR and/or the participant will be informed of the participant's inclusion in the trial. The study treatment period for the trial is limited i.e most participants will have completed all active treatment within 6 hours of randomisation. A smaller number of participants may receive active study treatment for up to a maximum of 24 hours. Therefore, the consent will be for the use of study data and follow up.

Dependent on local jurisdictional requirements and legislation, this trial will employ either one of the following 2 approaches:

- Opt-out
- Consent to continue

11.6.1 Opt-out approach

Subject to local HREC approval and where opt-out approach to consent is consistent with local jurisdictional requirements and legislation, we will request an opt-out approach for use of data and follow up. We believe the conditions for this opt-out approach as described in the National Statement 2.3.5 and 2.3.6 have been met in the ARISE Fluids trial. Refer to Appendix 1 for the National Statement 2.3.6 opt-out approach criteria and the trial's specific meeting of these conditions. Given our experience with trials in critically ill patients and from our consumer representative advice, when a waiver for enrolment has been granted, participants and LARs often find it confusing to provide written consent for data when the research treatment procedures have already taken place and they frequently perceive that more research specific interventional procedures are required.

Where an opt-out approach is approved, the site Principal Investigator, or their nominated delegate, will provide a plain language information sheet and brochure to the participant and/or LAR at the earliest appropriate opportunity. The information sheet and brochure will explain all aspects of the trial and the procedure to decline or opt-out from data collection and follow up. Additional to this written information, the site Principal Investigator, or their nominated delegate, will provide a verbal explanation of the trial and the opportunity to ask questions and have these answered. This combined process of both written information and verbal exchange will consistute an overall process to facilitate participant/LAR understanding of the information. It will also provide the best opportunity for the participant/LAR's understanding of the information to be demonstrated to the research member undertaking the opt-out approach. This information sheet and brochure will detail the process for declining or opting out of study participation. They will be given the information sheet and brochure

to keep and may seek further information or opt-out at anytime. The provision of the opt-out information sheet and brochure to the LAR/participant will be documented in the medical record and a copy of the information sheet and brochure will be placed in the participant's medical record. If the LAR or participant decides to opt-out of the trial this will be clearly documented in the medical record.

11.6.2 Consent to Continue

Subject to local HREC approval, and where opt-out consent is not consistent with local jurisdictional requirements and legislation, we will seek consent to continue participation in the trial. The site Principal Investigator, or their nominated delegate, will provide a plain language information sheet and consent form to the participant and/or LAR at the earliest appropriate opportunity. The information sheet will explain all aspects of the trial and the ability to decline or withdraw from data collection and follow up. Additional to this written information, the site Principal Investigator, or their nominated delegate, will provide a verbal explanation of the trial and the opportunity to ask questions and have these answered. This combined process of both written information and verbal exchange will constitute an overall process to facilitate participant/LAR understanding of the information. It will also provide the best opportunity for the participant/LAR's understanding of the information to be demonstrated to the research member undertaking the consent to continue. They will be given the information and consent form to keep and may seek further information or withdraw at anytime. The consent process will be documented in the medical record and a copy of the information and consent form will be placed in the participant's medical record.

11.6.3 Deceased patients

Participants enrolled in this study have septic shock and consequently the participant may deteriorate rapidly and unexpectedly. In the circumstance where a participant enrolled in the study under the process explained in 11.6.1 & 11.6.2 above, dies before consent has been obtained, we will use participants' data for the study. All attempts to contact the family and relevant circumstances prior to the death of any participant will be documented in the medical record. To seek consent from grieving family members would be stressful and inappropriate. Additionally, the study integrity and safety data would be compromised without this data.

11.6.4 Informed consent cannot be obtained from the participant or legally authorised representative

There may be the circumstance where a participants never regains competence following enrolment into the trial under the process explained in 11.6.2 above and there is no legally authorised representative available. If the participant is under a Guardianship Order then consent will be sought following the legislation and processes in place in that jurisdiction as long as the Guardianship Order includes consent to medical treatment. In the circumstance of a participant who never gains capacity and there is no LAR or a Guardianship Order which does not include decisions on consent to medical treatment, an approach will be made to the relevant HREC to request that study data may be retained and used.

11.7 Informed consent procedures- New Zealand context

In New Zealand, the approach used will be consistent with Right 7.4 of the Health and Disability Code [42], which outlines the framework for providing treatment to participants who are unable to consent for themselves. Consent will be in line with section 11.6.2 Consent to Contine with the addition of the specific approach detailed below.

The specific approach will be:

1. To consider whether the study assigned administration method and study participation is in the best interest of each individual patient, and

2. As soon as it is practical and reasonable, to seek the advice of persons interested in the participant's welfare to establish that study participation is consistent with the participant's wishes.

All participants who recover sufficiently will be given the opportunity to provide informed consent for ongoing study participation and for the use of data collected for the study.

12.DATA MANAGEMENT

12.1 Data collection and management methods

Data collection will be restricted to those variables necessary to define baseline participant characteristics, the delivery of the intervention in both arms, potential confounding co-interventions, and outcomes.

All data will be collected by trained research staff at each participating site; the paper case report form (CRF) worksheet may be used to collect the data from the medical record. Each participant will be assigned a unique study number at randomisation and only non-identifying data will then be entered into a web database (electronic case record form [eCRF]) by the site research staff. Data queries will be automatically generated via the electronic data collection database.

Randomised participants will be followed up until death or 90 days post-randomisation whichever occurs first for the primary outcome and thereafter up to 12 months for secondary outcomes. Follow up will be performed by site study staff by either direct contact with the participant or their LAR (if the participant has not regained adequate capacity). In all cases where the participant regains capacity they will be responsible for the provision of their own data. References to the LAR is only relevant where the participant has not regained capacity. Full protocol data will be collected for all randomised participants. If consent for participation is withdrawn or if the participant or LAR wish to opt-out, data will not be included unless consent to do so is obtained.

Participants and/or their LAR will be asked to provide three possible points of contact (home and close family contact details) to the research staff prior to discharge. Participants (or a LAR proxy) who are alive at 6 and 12 months after randomisation will be contacted by the site research coordinator via telephone. If it is not possible to contact the participant or LAR on the telephone, a copy of the relevant questionnaires will be sent to them via post or email. If the questionnaires are completed at home a return address, stamped-addressed envelope

will be provided. The research coordinators will administer, or the participants or LAR will complete, the WHODAS functional status assessment and EQ-5D-5L quality of life assessment.

12.2 Data variables collected

Data to be collected will include:

1. **Screening**
   - Patient identifier(s)
   - Inclusion and exclusion criteria
   - Reason for non-enrolment

2. **Baseline (immediately prior to randomisation)**
   - Baseline demographics (age, sex, weight, ethnicity)
   - Suspected source of infection
   - Usual residence
   - Pre-existing comorbidities (as defined by the Charlson Comorbidity Index Score)
   - Physiological and laboratory variables, if available
   - Date, time and name of first IV antimicrobial agent
   - IV fluids administered prior to randomisation (volume and type)
   - Severity of illness and organ failure scores (APACHE II, SOFA)

3. **During delivery of the intervention (up to 24 hrs)**
   - Physiological and laboratory variables, if available
   - Type and volume of IV fluids
   - Type and dose of vasoactive agents
4. **Other data**
   - Daily fluid balance until day 7 post-randomisation or ICU discharge whichever occurs first
   - Source of infection and microbial culture results
   - Type and timing of IV antimicrobials
   - Type and timing of co-interventions e.g. source control procedure, corticosteroids, extracorporeal membrane oxygenation
   - CVC insertion date and time
   - Complication outcomes (CVC complications, APO, ischaemic events)
   - Date and time consent granted or the date the study brochure presented
   - Treatment limitations or withdrawal
   - Patient clinical costings for index admission

5. **Adverse events**

See section 13 below

6. Outcome data

- Date/s and time of initiation and cessation of invasive ventilation
- Date/s and time of initiation and cessation of vasopressor support
- Date/s and time of initiation and cessation of acute renal replacement therapy
- Vital status at ICU, hospital discharge, day 28, day 90, 6 months and 12 months post-randomisation
- ED, ICU and hospital admission and discharge dates and times, including readmissions
- ED, ICU and hospital discharge destination
- Cause-specific hospital mortality
- Rehabilitation and residential care admission and discharge dates
- Functional status at 6- and 12-months post-randomisation (as measured by the WHODAS)
- Quality of life at 6- and 12-months post-randomisation (measured using the EQ-5D-5L)

12.3. Monitoring

Prior to study commencement, a start-up meeting will be held for all study staff. During the study, onsite [or remote as appropriate] and central monitoring will be conducted by the project manager or a delegated coordinating centre representative. A minimum of one monitoring and one close-out visit per site will occur. The purpose of these visits is to ensure the study is conducted according to the protocol, all applicable guidelines and regulations, and to perform source data verification.

Medical records, any other relevant source documents and the site investigator files must be made available to the monitor for these monitoring visits during the course of the study and at the completion of the study as needed.

A monitoring report will be prepared following each visit and reviewed by the management committee. A copy of the report will be sent to the principal investigator and study coordinator at the site and will be filed in the site investigator file.

The aims of monitoring visits are to:

- Check the accuracy of the data base by performing source data verification of the electronic CRF against the original source documents.
- Check for protocol deviations and report these to the chief investigator as necessary.
- Review outcome data for each monitored participant.
- Confirm the consent procedures approved by the site's HREC have been followed.
- Check data security and access.
- Review all serious adverse events (SAEs) and follow up all reported SAEs.
- Review investigator site files for completeness and accuracy.
- Assist the study staff with any queries or problems they may have in relation to the study.

12.4 Protocol deviations

A protocol deviation is an unanticipated or unintentional departure from the expected conduct of an approved trial that is not consistent with the current research protocol or consent document. A protocol deviation may be an omission, an error, addition or change in any procedure described in the protocol. If any ineligible participants are enrolled, as the study is intention to treat, these will be recorded as protocol deviations. Protocol deviations and rates of protocol deviations may be indicative of non-compliance with the protocol and will be regularly reported to the management committee. Reports on protocol deviations may trigger specific site contact and feedback and additional monitoring requirements/visits.

Given that the investigator is responsible for patient safety and care, he/she may implement a deviation from the protocol to eliminate an immediate hazard to trial participants without HREC approval. All protocol deviations must be reported via the trial database to the coordinating centre. The relevant HREC will be notified of protocol deviations or serious breaches in accordance with the NHMRC framework according to each HREC's requirements.

13.STATISTICAL CONSIDERATIONS

Participants who do not receive the allocated intervention according to the parameters set in the allocated study arm will be analysed according to the allocated arm according to the intention to treat principle.

13.1 Sample size calculation

Based on the data from the ARISE trial [10], the mean (standard deviation) of DAOH-D90 was 60(31). Assuming a clinically important increase of 7 days in the vasopressors arm, a sample size of 950 will have 90% power to detect this difference with a type I error rate of 0.05 (including a 15% inflation factor to account for the non-parametric distribution). Allowing for a 5% drop out rate, we will recruit 1000 participants to the trial. A 7 day difference in DAOH-D90 is considered clinically important and is supported by consumers.

13.2 Statistical and analytical plan

The primary outcome (DAOH-D90) and other non-parametric outcomes will be analysed using quantile regression with results presented as median difference (95%CI). Mortality and other binomial outcomes will be analysed using logistic regression with results reported as odds ratios (95%CI). Time to event outcomes will be analysed using proportional hazards regression with results presented as Kaplan Meier or cumulative incidence curves where mortality is a competing risk. Functional status and quality of life will employ longitudinal analysis techniques to account for data collected at both 6 and 12 months. All analyses will account for site with participants nested within site and site treated as a random effect. Where baseline imbalance (p<0.2) is observed between treatment arms, sensitivity analysis will be performed adjusting for imbalanced variables. Sensitivity to missingness will be performed using multiple imputation. A secondary analysis will be conducted using a Bayesian approach to model a posterior probablility of the odds ratio for days alive and out of hospital at 90

days post-randomisation between the intervention and control group with assosciated 95% credible interval (CrI). Statistical analysis will be performed using SAS or R and a detailed statistical analysis plan will be published or posted in the public domain prior to study completion.

13.3 Subgroup analyses

The following subgroups will be analysed for the primary outcome:

1. Age <65 years vs ≥65 years
2. Sex
3. Lactate <3 mmol/L vs ≥3 mmol /L at baseline
4. APACHE II score <15 vs ≥15
5. Source of infection (respiratory, urinary, other)
6. Fluid volume prior to randomisation dichotomised at the median volume.

13.4 Interim analysis

Following the recruitment of the first 150 paticipants, a review will be undertaken of recruitment rates, protocol compliance and separation between the study treatment arms.

The DSMC will review a formal interim analysis performed at 50% participant enrolment.

14. SAFETY

14.1 Data Safety Monitoring Committee

An independent data safety monitoring committee (DSMC), comprising experts in clinical trials, biostatistics, emergency medicine and intensive care has been established before participant enrolment and will review all trial protocols. The committee will be charged with monitoring trial safety and efficacy and overseeing the interim analysis. The DSMC may, in its absolute discretion, request assessment of any other trial data at any time.

The project manager will forward a copy of all serious adverse event (SAE) reports as soon as they become available to the DSMC or at the discretion of the DSMC, their delegated independent safety monitor . The DSMC or their delegated independent safety monitor will review all SAE reports that are received and the DSMC may report back to the management committee of the study if any further action is required.

14.2 Adverse events

Adverse events (AEs) are defined as any untoward medical occurrence in a patient or clinical investigation participant administered an investigational intervention and which does not necessarily have to have a causal relationship with this treatment (adapted from the Note for Guidance on Clinical Safety Data Management: Definitions and Standards for Expedited Reporting (CPMP/ICH/377/95 July 2000).

It is recognised, however, that the participant population with severe sepsis in the ED and ICU will experience a number of common aberrations in laboratory values, signs and symptoms due to the severity of the underlying disease and the impact of standard therapies. These will not necessarily constitute an adverse event unless they require significant intervention and are deemed to be causally related to the study intervention arms (possibly, probably or definitely) by the investigator or are considered to be of concern in the investigator's clinical judgement. Adverse events already defined as study complication outcomes (e.g. CVC complications, acute pulmonary oedema, vasopressor complications) will not be reported separately as adverse events to facilitate unbiased reporting. In all cases, the condition or disease underlying the symptom, sign or laboratory value should be reported e.g. pulmonary oedema rather than bilateral inspiratory auscultatory crackles.

14.3 Serious adverse events

The baseline mortality of participant with septic shock is high. Such patients will frequently develop life-threatening organ failure(s) unrelated to the study interventions and despite optimal management. Therefore, events that are part of the natural history of the disease process or are expected complications of septic shock will not be reported as serious adverse events in this study. In particular, events already defined as study outcomes (e.g. death, readmission) will not be reported separately as serious adverse events unless they are considered to be causally related to the study intervention or are otherwise a concern in the investigator's judgement.

SAEs will be defined in accordance with the Note for Guidance on Clinical Safety Data Management: Definitions and Standards for Expedited Reporting (CPMP/ICH/377/95) (July 2000) as any untoward medical occurrence that:

- Results in death
- Is life-threatening
- Requires inpatient hospitalisation or prolongation of existing hospitalisation
- Results in persistent or significant disability/incapacity
- Is a congenital anomaly/birth defect
- Is an important medical event which may require intervention to prevent one of the previously listed outcomes

14.4 Reporting

AEs and SAEs, which are deemed causally related to the study intervention by the investigator, will be collected from randomisation up to hospital discharge.

SAEs will be reported to the coordination centre within 24 hours of the site investigator becoming aware via completion of the SAE form on the website. Minimum data will include the patient study ID, nature of the event, commencement and cessation of the event, the investigators opinion of the relationship of the event to the trial

(possibly, probably or definitely related), and any treatment which was required. The investigator should notify the Institutional/Ethics Committee of the occurrence of the serious adverse event or any significant safety issues in accordance with local jurisdictional requirements/policy directives.

15.FUNDING

The ARISE FLUIDS trial is funded by the Medical Research Future Fund Rare Cancers, Rare Disease and Unmet Need grant scheme (APP1200084).

16.PUBLICATION POLICY

All publications arising from the trial will comply with the requirements of the ANZIC-RC, the ANZICS CTG and ACEM CTN for group authorship. Publications will be authored on “behalf of the ARISE FLUIDS Investigators”. A writing committee will be convened from the membership of the management committee. Authors named in the journal by-line will meet the authorship criteria defined by the International Committee of Medical Journal Editors (ICJME). Membership of the ARISE FLUIDS Investigators group will comprise site investigators and other contributors and will be listed in the acknowledgement section of the paper. These will be designated contributors to the study in PubMed.

Funding bodies will be acknowledged in the publication.

17.TRIAL REGISTRATION

This trial has been registered on clinical trials.gov NCT 04569942.

CONFIDENTIAL

18.REFERENCES

1. Reinhart, K., et al., *Recognizing sepsis as a global health priority—a WHO resolution.* New England Journal of Medicine, 2017. **377**(5): p. 414-417.
2. Rudd, K.E., et al., *Global, regional, and national sepsis incidence and mortality, 1990–2017: analysis for the Global Burden of Disease Study.* The Lancet, 2020. **395**(10219): p. 200-211.
3. Thompson, K., et al., *Health-related outcomes of critically ill patients with and without sepsis.* Intensive Care Medicine, 2018. **44**(8): p. 1249-1257.
4. Finfer, S., et al., *Adult-population incidence of severe sepsis in Australian and New Zealand intensive care units.* Intensive Care Medicine, 2004. **30**(4): p. 589-596.
5. Kaukonen, K.-M., et al., *Mortality related to severe sepsis and septic shock among critically ill patients in Australia and New Zealand, 2000-2012.* JAMA, 2014. **311**(13): p. 1308-1316.
6. Prescott, H.C. and D.C. Angus, *Enhancing recovery from sepsis: a review.* JAMA, 2018. **319**(1): p. 62-75.
7. Yende, S., et al., *Long-term quality of life among survivors of severe sepsis: analyses of two international trials.* Critical Care Medicine, 2016. **44**(8): p. 1461.
8. Singer, M., et al., *The third international consensus definitions for sepsis and septic shock (Sepsis-3).* JAMA, 2016. **315**(8): p. 801-810.
9. Rhodes, A., et al., *Surviving sepsis campaign: international guidelines for management of sepsis and septic shock: 2016.* Intensive Care Medicine, 2017. **43**(3): p. 304-377.
10. ARISE Investigators and the ANZICS Clinical Trials Group, *Goal-directed resuscitation for patients with early septic shock.* New England Journal of Medicine, 2014. **371**(16): p. 1496-1506.
11. Acheampong, A. and J.-L. Vincent, *A positive fluid balance is an independent prognostic factor in patients with sepsis.* Critical Care, 2015. **19**(1): p. 251.
12. Marik, P.E., et al., *Fluid administration in severe sepsis and septic shock, patterns and outcomes: an analysis of a large national database.* Intensive Care Medicine, 2017. **43**(5): p. 625-632.
13. Maitland, K., et al., *Mortality after fluid bolus in African children with severe infection.* New England Journal of Medicine, 2011. **364**(26): p. 2483-2495.
14. Maitland, K., et al., *Exploring mechanisms of excess mortality with early fluid resuscitation: insightsfrom the FEAST trial.* BMC medicine, 2013. **11**(1): p. 1-15.
15. Andrews, B., et al., *Effect of an early resuscitation protocol on in-hospital mortality among adults with sepsis and hypotension: a randomized clinical trial.* JAMA, 2017. **318**(13): p. 1233-1240.
16. Angus, D.C. and T. Van der Poll, *Severe sepsis and septic shock.* New England Journal of Medicine, 2013. **369**: p. 840-851.
17. van Ierssel, S.H., et al., *The endothelium, a protagonist in the pathophysiology of critical illness: focus on cellular markers.* BioMed Research International, 2014. **2014**.
18. Burke-Gaffney, A. and T.W. Evans, *Lest we forget the endothelial glycocalyx in sepsis.* Critical Care, 2012. **16**(2): p. 121.
19. Chappell, D., et al., *Hypervolemia increases release of atrial natriuretic peptide and shedding of the endothelial glycocalyx.* Critical Care, 2014. **18**(5): p. 538.

20. Byrne, L., et al., *Unintended consequences: fluid resuscitation worsens shock in an ovine model of endotoxemia.* American Journal of Respiratory and Critical Care Medicine, 2018. **198**(8): p. 1043-1054.
21. Silversides, J.A., et al., *Conservative fluid management or deresuscitation for patients with sepsis or acute respiratory distress syndrome following the resuscitation phase of critical illness: a systematic review and meta-analysis.* Intensive Care Medicine, 2017. **43**(2): p. 155-170.
22. Hjortrup, P.B., et al., *Restricting volumes of resuscitation fluid in adults with septic shock after initial management: the CLASSIC randomised, parallel-group, multicentre feasibility trial.* Intensive Care Medicine, 2016. **42**(11): p. 1695-1705.
23. Meyhoff, T.S., et al., *Lower versus higher fluid volumes during initial management of sepsis–a systematic review with meta-analysis and trial sequential analysis.* Chest, 2020;157(6):1478-1496.
24. Li, Y., H. Li, and D. Zhang, *Timing of norepinephrine initiation in patients with septic shock: a systematic review and meta-analysis.* Critical Care, 2020. **24**(1): p. 1-9.
25. Myles, P.S., et al., *Restrictive versus liberal fluid therapy for major abdominal surgery.* New England Journal of Medicine, 2018. **378**(24): p. 2263-2274.
26. Macdonald, S.P. and N.I. Shapiro, *Liberal or restricted fluid resuscitation in critical illness: Shifting the needle back towards equipoise.* Emergency Medicine Australasia, 2018. **30**(4): p. 446-447.
27. ProCESS Investigators, *A Randomized Trial of Protocol-based Care for Early Septic Shock.* American Journal of Respiratory and Critical Care Medicine, 2014. **190**(7): p. 827.
28. Prism Investigators, *Early, goal-directed therapy for septic shock—a patient-level meta-analysis.* New England Journal of Medicine, 2017. **376**(23): p. 2223-2234.
29. Keijzers, G., et al., *The Australasian Resuscitation In Sepsis Evaluation: Fluids or vasopressors in emergency department sepsis (ARISE FLUIDS), a multi-centre observational study describing current practice in Australia and New Zealand.* Emergency Medicine Australasia, 2020. **32**(4): p. 586-598.
30. Udy, A.A., et al., *Incidence, patient characteristics, mode of drug delivery, and outcomes of septic shock patients treated with vasopressors in the arise trial.* Shock, 2019. **52**(4): p. 400-407.
31. Delaney, A., et al., *Initiation of vasopressor infusions via peripheral versus central access in patients with early septic shock: A retrospective cohort study.* Emergency Medicine Australasia, 2020. **32**(2): p. 210-219.
32. Tian, D.H., et al., *Safety of peripheral administration of vasopressor medications: A systematic review.* Emergency Medicine Australasia, 2020. **32**(2): p. 220-227.
33. Macdonald, S.P., et al., *Restricted fluid resuscitation in suspected sepsis associated hypotension (REFRESH): a pilot randomised controlled trial.* Intensive Care Medicine, 2018. **44**(12): p. 2070-2078.
34. Myles, P.S., et al., *Validation of days at home as an outcome measure after surgery: a prospective cohort study in Australia.* BMJ Open, 2017. **7**(8).
35. Fanaroff, A., et al., *Days Alive and Out of Hospital: Exploring a Patient-Centered, Pragmatic Outcome in a Clinical Trial of Patients With Acute Coronary Syndromes.* Circulation: Cardiovascular Quality and Outcomes, 2018. **11**(12).

36. Hodgson, C.L., et al., *Treatment of invasively ventilated adults with Early Activity and Mobilisation*. 2017, Monash University.
37. Civil and Administrative Tribunal New South Wales, Shehabi v Attorney General (NSW) [2016] NSWCATAP 137. 2016, NSW Caselaw.
38. Peake, S.L., et al., Australasian resuscitation of sepsis evaluation (ARISE): A multi-centre, prospective, inception cohort study. Resuscitation, 2009. 80(7): p. 811-818.
39. Keijzers, G., et al., The Australasian Resuscitation In Sepsis Evaluation: Fluids or vasopressors in emergency department sepsis (ARISE FLUIDS), a multi-centre observational study describing current practice in Australia and New Zealand. Emergency Medicine Australasia, 2020. 32(4): p. 856-598.
40. Clinical Excellence Commission, Sepsis Pathways, 2024. CEC Health NSW.
41. National Health and Medical Research Council, *National statement on ethical conduct in human research*, in *National Statement on Ethical Conduct in Human Research 2007*, the Australian Research Council and the Australian Vice-Chancellors’ Committee, Editor. 2018, Commonwealth of Australia, : Canberra.
42. New Zealand Health and Disability Commissioner, *Code of Health and Disability Services Consumers' Rights*. 1996.

19.APPENDIX 1: OPT-OUT APPROACH AND THE NATIONAL STATEMENT

This trial meets the conditions for an opt- out approach as detailed in the National statement 2.3.6 as follows:

a) involvement in the research carries no more than low risk to participants

This trial where both trial arms are encompassed by the range of usual care in Australia and New Zealand, confers no greater clinical risk than receiving routine treatment. Refer to protocol section 11.3.

b) the public interest in the proposed activity substantially outweighs the public interest in the protection of privacy

This trial will address an important gap in the evidence base and has the potential to provide future guidance for bedside clinicians and optimise important patient-centred outcomes. As such it has significant public interest while privacy is protected by an opt-out approach and study design methods. Refer to protocol section 11.4.

c) *the research activity is likely to be compromised if the participation rate is not near complete, and the requirement for explicit consent would compromise the necessary level of participation*

Participants (when deemed competent) and/or their LAR will be approached as soon as reasonably possible after enrolment. An opt-out approach will maintain the right to voluntarily determine participation while easing participant/LAR burden in indicating that decision which explicit consent imposes. Refer to protocol section 11.6.1.

d) reasonable attempts are made to provide all prospective participants with appropriate plain language information explaining the nature of the information to be collected, the purpose of collecting it, and the procedure to decline participation or withdraw from the research

All participants and/or their LAR will be provided with written and verbal information to facilitate understanding of all aspects of the trial and the procedure for declining participation. Refer to protocol section 11.6.1.

e) a reasonable time period is allowed between the provision of information to prospective participants and the use of their data so that an opportunity for them to decline to participate is provided before the research begins

Participants and/or their LAR will be approached at the earliest reasonable opportunity to consider their ongoing participation in the trial. They will have the ability to decline participation at this point or at any future time through the mechanism described in the information sheet/ brochure. As this encompasses the use of data and follow up telephone calls at day 90 and at 6 and 12 months they can decline/withdraw from further participation at any time before these time points.

f) a mechanism is provided for prospective participants to obtain further information and decline to participate

The information sheet/brochure, as approved by the relevant ethics committee, will contain contact numbers/details on who can provide further information and how to decline participation. Refer to protocol section 11.6.1.

g) the data collected will be managed and maintained in accordance with relevant security standards

The trial will adhere to all relevant data collection security standards, including those of Monash University as the trial coordinating centre. Refer to protocol section 12.

h) there is a governance process in place that delineates specific responsibility for the project and for the appropriate management of the data

The specific responsibilities for the trial itself and the appropriate data management are detailed in protocol sections 3 and 12.

i) the opt-out approach is not prohibited by state/territory, federal, or international law.

The opt-out approach is one of the 2 possible approaches to consent this study may use. Where opt-out approach is not possible due to local state/territory laws, then consent will be sought as consent to continue as detailed in protocol section 11.6.2.
