# Supplementary material for: Australasian Resuscitation In Sepsis Evaluation: FLUid or vasopressors In emergency Department Sepsis (ARISE FLUIDS) trial: study protocol
Source: BMJ Open. 2025 Jul 20;15(7):e101215. doi: 10.1136/bmjopen-2025-101215 (PMC12278162; doi:10.1136/bmjopen-2025-101215)
Supplement: online supplemental file 4 [file bmjopen-15-7-s004.pdf]

Appendix 4. Summary of data to be collected

**Time of collection**	**Data collected**
Patient screening and eligibility	Site name, number and patient identifiers<br>ED triage date and time<br>Inclusion and exclusion criteria, date and time<br>Randomisation date and time and treatment allocation
Baseline patient characteristics	Demographics (age, sex, weight, ethnicity)<br>Usual residence[#]<br>Chronic co-morbidities and functional status*<br>Severity of illness and organ failure scores (APACHE II, SOFA)<br>Physiological and laboratory variables^<br>Total IV fluids administered prior to randomisation[+]
Intervention delivery (T0-24 hrs)	Physiological and laboratory variables, T6, T12, T18, T24 hrs<br>Hourly type and volume IV fluids, T0-T6 hrs[&]<br>6-hourly type and volume IV fluids, T6-T24 hrs<br>6-hourly type, dose and route vasopressors, T0-T24 hrs<br>CVC or PICC insertion, date and time<br>Compliance with study intervention[##]
Microbiology (up to T72 hrs)	Source of infection[!]<br>Positive microbial results e.g., cultures, serology, PCR<br>Type and timing IV antimicrobials<br>Appropriateness of antimicrobial therapy[!]
During hospital admission	Study brochure presented or consent granted, date and time<br>Daily fluid balance in ICU days 1-7 post-randomisation<br>Co-interventions e.g., source control, corticosteroids<br>Peripheral vasopressor complications e.g., tissue necrosis<br>CVC or PICC complications e.g., pneumothorax, infection

	Complications e.g., APO, cardiac, digital, intestinal ischaemia[%]<br>Invasive/non-invasive ventilation, all episodes, date and time<br>Vasopressor administration, all episodes, date and time<br>Renal replacement therapy, all episodes, date and time<br>ED, ICU and hospital admission and discharge, date and time<br>ICU readmissions and discharge, date and time<br>Treatment limitations or withdrawal<br>ED, ICU and hospital discharge destination<br>Vital status at ICU and hospital discharge<br>Cause-specific hospital mortality<br>Adverse events, type, timing, grading, causality and resolution<br>Patient clinical costings for index hospital admission
Up to day 90 post-randomisation	Vital status and date of death, if applicable<br>Hospital readmission and discharge dates, all episodes<br>Rehabilitation, long-term care admission and discharge dates
6 and 12 mths post-randomisation	Vital status and date of death, if applicable<br>Quality of life using EQ-5D-5L questionnaire<br>Functional status using WHODAS 2.0 questionnaire

IV denotes intravenous; APACHE, Acute Physiology and Chronic Health Evaluation, SOFA, Sequential Organ Failure Assessment; Hrs, hours; CVC, central venous catheter; PICC, peripherally inserted central catheter; PCR, polymerase chain reaction; ECMO, extracorporeal membrane oxygenation; APO, acute pulmonary oedema; ED, emergency department; ICU, intensive care unit; mths, months WHODAS, World Health Organisation Disability Assessment Schedule

# Usual residence categorised as home or long-term care facility. Home includes independent living in a retirement village, hospital in the home and independent hostel or rest home.

* Co-morbidities defined using the Charlson Comorbidity Index. Functional status categorised as independent with all activities of daily living (ADL) or requires help with ≥ 1 ADL (e.g., dressing, bathing).

^ Data, when available, includes vital signs, non-sedated Glasgow Coma Score, creatinine, bilirubin, lactate, haematocrit, white cell count, platelet count, arterial blood gas analysis.

+ Includes IV fluid volume administered up to the time final inclusion criterion met and, to account for ongoing fluid resuscitation during the randomisation process, the IV fluid volume administered between meeting the final inclusion criterion and randomisation.

& IV fluids include crystalloids, colloids and blood products.

@ Data, when available includes vital signs, saturation of peripheral oxygen, lactate and arterial blood gas analysis.

! Assessment performed by site Principal Investigator at resolution of septic shock or death, whichever occurs first.

Assessment of compliance includes: 1) enrolment of ineligible patient; 2) fluid arm, fluid bolus not administered for hypotension and; 3) vasopressor arm, vasopressor not commenced for hypotension and/or hypoperfusion, fluid bolus administered without a study-specific indication and fluid bolus > 250 mL administered

% Collected on all patients irrespective of treatment allocation and, in the opinion of the Principal Investigator, causally and/or temporally related to the study intervention.
