# Supplementary material for: Australasian Resuscitation In Sepsis Evaluation: FLUid or vasopressors In emergency Department Sepsis (ARISE FLUIDS) trial: study protocol
Source: BMJ Open. 2025 Jul 20;15(7):e101215. doi: 10.1136/bmjopen-2025-101215 (PMC12278162; doi:10.1136/bmjopen-2025-101215)

The ARISE FLUIDS Study

Data Safety Monitoring Committee Charter

Title of the protocol:	**Australasian Resuscitation In Sepsis Evaluation: FLUid or vasopressors In emergency Department Sepsis**

Protocol Number:	ANZIC-RC/SP002
Clinical Trial Registration:	ClinicalTrials.gov NCT04569942
Coordinating centre:	The Australian and New Zealand Intensive Care Research Centre, Monash University<br>Level 3, 553 St. Kilda Rd<br>Melbourne, Victoria 3004 Australia
Version	1
Date of Document	10 May 2021

Change Control Register		
Version	Date of Document	Approved by
1	10 May 2021	DSMC

Table of contents

1. Introduction

2. The DSMC

2.1 Independence of the DSMC

2.2 Primary role and responsibilities of the DSMC

2.3 Conflicts of Interest

2.4 Membership of the DSMC

3. Trial Statistician

4. Organisational Structure Diagram

5. The Trial Management Committee role in relation to the DSMC

6. Timing and Purpose of the DSMC Meetings

6.1 Organisational Meeting

6.2 Preliminary Data Review Meeting

6.3 Interim Analysis

6.4 Additional Data Reporting

7. Adverse Event reporting

8. Serious Adverse Event reporting

9. Study outcome reporting

10. Procedures to Ensure Confidentiality and Proper Communication

11. Closed session

12. Open Session

13. Open and Closed Reports

14. Minutes of the DSMC Meeting

15. Recommendations to the Management Committee (MC)

16. Statistical Monitoring Guidelines

Figure 1. ARISE FLUIDS trial organisational structure diagram

1. Introduction

This Charter is for the Data Safety Monitoring Committee (DSMC) of the ARISE FLUIDS trial.

The purpose of this document is to describe the roles and responsibilities of the independent DSMC, the DSMC membership, its relationship with the trial's other committees, and the purpose, format and timing of its meetings. The Charter will also detail the procedures for ensuring trial confidentiality and proper communication and an outline of the content of the Open and Closed Reports that will be provided to the DSMC. The statistical monitoring guidelines to be implemented by the DSMC will be detailed in this charter.

This DSMC charter conforms with the National Health and Medical Research Council's Guidance on Data Safety Monitoring Boards 2018.

2. The DSMC

2.1 Independence of the DSMC

The DSMC is a separate entity from the ARISE FLUIDS Management Committee and its members are fully independent of the sponsoring institution, Monash University, and all trial investigators. The DSMC functions to provide expert and independent advice to the trial's Management Committee, so that jointly safety of trial participants and data integrity can be assured. The independence of the DSMC is intended to control the sharing of important comparative information necessary to oversee patient safety and data integrity while safeguarding the integrity of the clinical trial from any adverse impact resulting from access to trial information.

2.2 Conflicts of Interest

The DSMC membership has been restricted to individuals free of any competing interest that may impact on the trial. The nature of a competing interest may be financial, scientific or regulatory in nature. Any competing interest or lack thereof, which may be real or potential should be declared in writing.

Any DSMC member who develops potentially relevant conflicts of interest during the course of the trial should promptly bring these to the attention of the DSMC chairperson for a decision by the remaining committee members as to the individual's ongoing eligibility to function within the committee. The DSMC will be responsible for deciding whether these competing interests materially impact objectivity.

DSMC membership is to be for the duration of the clinical trial. If any members leave the DSMC during the course of the trial, the DSMC will promptly appoint their replacements of equivalent expertise.

2.3 Primary role and responsibilities of the DSMC

The DSMC will be responsible for safeguarding the interests of trial participants, assessing the safety and efficacy of the interventions during the trial, and for monitoring the overall conduct of the clinical trial.

The DSMC will review the trial's progress and accruing data of the trial at scheduled interim analysis point and at any other time the DSMC deems necessary. This includes but is not limited to, accumulated trial data on:

- recruitment,
- data quality,
- protocol compliance
- main outcome/s and
- safety data.

Based on this review, the DSMC may make recommendations to the ARISE FLUIDS trial Management Committee. As a primary responsibility, the DSMC will consider and make an assessment of trial safety. The DSMC may, based on their review of the trial's scientific, medical, and ethical criteria, make recommendations to the ARISE FLUIDS trial Management Committee regarding the:

- continuation without modifications,
- continuation with modifications,
- temporary pause in recruitment, or
- termination of the study.

At their discretion, the DSMC may also formulate recommendations relating to the protocol or protocol changes such as the selection, recruitment, and retention of participants, their management, improving adherence to protocol-specified regimens and the procedures for data management and quality control.

The DSMC serves in an advisory role to the ARISE FLUIDS Management Committee in regards to protocol amendments. Thus, the DSMC will be consulted regarding protocol changes but will not be responsible for providing formal approval of protocol changes

The DSMC will review external information that may have an impact on the study.

In line with standard research practice in Australia, there is no formal reporting by the DSMC to the National Health and Medical Research Council (NHMRC).

2.4 Membership of the DSMC

The DSMC is a fully independent multidisciplinary group who collectively have experience in:

- Emergency and Intensive care medicine and research

- Biostatistics

- Conduct, analysis and monitoring of randomised controlled trials
- Previous experience on DSMC.

**DSMC Chair:**	[redacted]
**DSMC Members:**	[redacted]<br>Emergency Medicine,<br>[redacted]
	[redacted]<br>Critical Care and Emergency Medicine,<br>[redacted]
	[redacted]
	[redacted]<br>Senior Statistician,<br>[redacted]

3. Trial Statistician

The trial statistician, who is independent of the trial management committee, will have access to all trial data and will prepare the data and reports for the DSMC at the preliminary data review meeting and interim analysis and at any other time point at the DSMC's request. In this role he will attend the closed session of the DSMC however will have no role on the DSMC.

Trial Statistician	[redacted]

4. The Trial Management Committee role in relation to the DSMC

The ARISE FLUIDS Management Committee will be responsible for promptly reviewing the DSMC recommendations.

In the event the DSMC raises any concerns related to the study protocol or any proposed protocol changes, the ARISE FLUIDS Management Committee will consider and discuss these and to seek to achieve an outcome by mutual agreement between the two Committees.

The ARISE FLUIDS trial Management Committee, however, will be ultimately responsible for deciding whether to continue, modify or to stop the trial based on the DSMC recommendations and to determine whether amendments to the protocol or changes in study conduct are required.

It will be the responsibility of the ARISE FLUIDS Management Committee to inform the DSMC of the results of related trials that conclude during the course of the ARISE FLUIDS trial or other information that arises that may impact on the study.

5. Organisational Structure Diagram

The organizational diagram shows the relationship between the DSMC and other committees and functional areas/data flow involved in the trial. Refer to Figure 1.

6. Timing and Purpose of the DSMC Meetings and Reports

6.1 Initial Organisational Meeting

The initial meeting of the DSMC will be an 'Organisational Meeting' which is held before the start of trial recruitment. This Organisational Meeting will include the DSMC chair and members, the trial Chief Investigators, project manager and the trial statistician.

This meeting will provide opportunity for advisory review of scientific and ethical issues relating to study design and conduct, discuss the role and functioning of the DSMC and the format and content of the Open and Closed Reports that will be used to present trial results at future DSMC meetings.

This organisational meeting may occur via teleconference or through email exchange.

The DSMC Chair will be provided with the current copies of the clinical trial protocol, the DSMC Charter, and the current versions of the case report forms.

6.2 Preliminary Data Review meeting

Following the recruitment of the first 150 participants, the DSMC will review the following:

- Recruitment, including screening data, enrolment by site/month
- Protocol compliance, including protocol deviationsand
- Separation between the study treatment arms.

The accompanying DSMC tables document details the data to be presented to the committee at the preliminary data review and interim analysis meetings.

At the DSMC's discretion other variables can be reviewed as deemed necessary.

6.3 Interim Analysis

One interim analysis is planned for the ARISE FLUIDS Study. This will be performed after recruitment of 50% of the 1000 target number of patients recruited have reached the 90 day outcome. A cut-off date one month after the 500th patient reaches 90 day follow up will be set so the interim analysis is conducted in a timely manner.

The independent trial statistician will prepare the blinded data for the DSMC for discussion at a formal closed meeting. Data prepared for the interim analysis should be available to the DSMC members in advance of the meeting. Data will be analysed according to group allocation. All data will remain confidential to the DSMC and the independent trial statistician.

A teleconference attended by all members of the DSMC and the trial statistician, will be organised as soon as possible after the 90-day follow up censor period of one month and data are analysed. A consensus decision of the DSMC will be required to make a recommendation regarding cessation of the trial. In the event a consensus decision cannot be reached, subsequent interim analyses may be undertaken prior to further consideration of early cessation.

6.3 Additional Data Reporting

The DSMC can request any additional data at other intervals at their discretion.

7. Adverse Event (AE) reporting

Adverse events (AEs) are defined as any untoward medical occurrence in a patient or clinical investigation participant administered an investigational intervention and which does not necessarily have to have a causal relationship with this treatment (adapted from the Note for Guidance on Clinical Safety Data Management: Definitions and Standards for Expedited Reporting (CPMP/ICH/377/95 July 2000).

It is recognised, however, that the participant population with severe sepsis in the ED and ICU will experience a number of common aberrations in laboratory values, signs and symptoms due to the severity of the underlying disease and the impact of standard therapies. These will not necessarily constitute an adverse event unless they require significant intervention and are deemed to be causally related to the study intervention arms (possibly, probably or definitely) by the investigator or are considered to be of concern in the investigator's clinical judgement. Adverse events already defined as study complication outcomes (e.g. CVC complications, acute pulmonary oedema) will not be reported separately as adverse events to facilitate unbiased reporting.

AEs which are deemed causally related to the study intervention by the investigator, will be collected from randomisation up to hospital discharge.

AEs decided to be possibly, probably or definitely causally related to study procedures will be reported to the coordinating centre staff and recorded in database. Non-serious adverse events will not be individually notified to the DSMC or site HRECs in accordance with local practice. However, the DSMC will have free access to the safety database information at all times upon request and may make recommendations for the ongoing conduct of the trial based on this information.

8. Serious Adverse Event (SAE) reporting

Serious Adverse Events (SAEs) are defined as any untoward medical occurrence that meets one or more of the following criteria:

- Results in death;
- Is life-threatening;
- Requires inpatient hospitalisation or prolongation of existing hospitalisation;
- Results in persistent or significant disability/incapacity, or disability/incapacity that is likely to become persistent or significant;
- Is a congenital anomaly/birth defect; or
- Is an important medical event which may require intervention to prevent one of the previously listed outcomes.

The baseline mortality of participant with septic shock is high. Such patients will frequently develop life-threatening organ failure(s) unrelated to the study interventions and despite optimal management. Therefore, events that are part of the natural history of the disease process or are expected complications of septic shock will not be reported as serious adverse events in this study. In particular, events already defined as study outcomes (e.g. death, readmission) will not be reported separately as serious adverse events unless they are considered to be causally related to the study intervention or are otherwise a concern in the investigator's judgement.

SAEs which are deemed causally related to the study intervention by the investigator, will be collected from randomisation up to hospital discharge.

SAEs should be reported to the coordinating centre, by entering into the study website, within 24 hours of study staff becoming aware of the event.

The Coordinating Centre will report each SAE to the chair of the DSMC or at the discretion of the DSMC, their delegated independent safety monitor. The DSMC or their delegated independent safety monitor will review all SAE reports that are received and may report back to the Management Committee of the trial if any further action is required.

9. Study outcomes reporting

The study CRF collects pre-specified complications as study outcomes. Study outcome events will not be reported separately as adverse events in order to facilitate unbiased reporting. Events already defined as study outcomes will not be reported separately as serious adverse events unless they are considered to be causally related to the study intervention or are otherwise a concern in the investigator's judgement.

Study complication outcomes which are collected in the CRF are:

- Peripheral administration vasopressor complications:
    - Tissue necrosis
    - Other complication
- Central venous catheter/peripherally inserted central catheter (CVC/PICC) related complication when insterted from T0-T24:
    - Pneumothorax
    - Arterial puncture
    - CVC related infection
    - CVC related thrombosis
    - Other complication
- Acute pulmonary oedema related to study intervention
- Ischaemic complications related to the study intervention:
    - Ischaemic bowel

- Ischaemic digits
- Cardiac ischaemia
- Other ischaemic complications

10. Procedures to ensure confidentiality and proper communication

To enhance the integrity and credibility of the trial, procedures will be implemented to ensure the DSMC has sole access to evolving information from the trial. An exception will be made to permit access to the independent trial statistician to blinded group allocation data. This will enable the trial statistician to serve as a liaison between the database and the DSMC. The study's project manager will have immediate access on an ongoing basis to patient-specific information on SAEs to satisfy the standard requirement for prompt reporting to the regulatory authorities.

At the same time, procedures will be implemented to ensure proper communication is achieved between the DSMC and the ARISE FLUIDS Management Committee. To provide a forum for exchange of information among various parties who share responsibility for the successful conduct of the trial, a format for Open Sessions and Closed Sessions will be implemented. The intent of this format is to enable the DSMC to preserve confidentiality of the comparative efficacy and safety results while at the same time providing opportunities for interaction between the DSMC and others who have valuable insights into trial-related issues.

Any recommendations made by the DSMC will be formally communicated to the Chief Investigator and the project manager in writing.

11. Closed Sessions

Sessions involving only DSMC members (Closed Sessions) will be held to allow discussion of confidential data from the clinical trial, including information about the safety of interventions. At the Preliminary Data Review Meeting the DSMC will review recruitment, protocol compliance and separation between the study treatment arms and report on this to the trial Management Committee. At the Interim Analysis (Closed Session), the DSMC will develop a consensus on its list of recommendations, including that relating to whether the trial should continue without modifications, continue with modifications, or stop.

The independent trial statistician will facilitate the discussion by explaining the analysis and responding to any statistical queries pertaining to the data. The members will then have time to discuss the confidential data from the clinical trial, including information about the efficacy and safety of interventions.

To ensure that the DSMC will be fully informed in its primary mission of safeguarding

the interest of participating patients and data integrity, the DSMC will receive data analysed with treatment group indicated.

12. Open Session

In order to allow the DSMC to have adequate opportunity to discuss the study generally, a joint session between DSMC members, the trial statistician and ARISE FLUIDS chief investigators and project manager (called an Open Session) will be held. This will occur before the Closed Session of the preliminary data review meeting and the interim analysis. A further open session can occur after the closed session, at the discretion of the DSMC.

Open sessions are not mandated at any additional sessions of the DSMC to review data reports, unless requested by the DSMC. Open session/s give the DSMC an opportunity to query these individuals about issues that have arisen during their review in the Closed Session. With this format, important interactions are facilitated through which problems affecting trial integrity can be identified and resolved.

13. Open and Closed Reports

For each DSMC meeting, Open and Closed reports will be provided.

Open Reports, available to all who attend the DSMC meeting, will include pooled data:

- on screening & recruitment
- eligibility
- completeness of data & follow-up
- compliance/protocol deviations
- data management, quality and monitoring.

The Open Reports will be prepared by the project manager.

Closed Reports, available only to those attending the Closed Sessions of the DSMC meeting, of data separated by group allocation will include:

- analyses of primary and secondary efficacy endpoints,
- compliance/protocol deviations,
- analyses of AEs, SAEs and safety endpoints including complication outcomes.

The Closed Reports will be prepared by the independent trial statistician.

The Open and Closed Reports should provide information that is accurate, with follow-up that is complete to within one month of the date of the DSMC meeting. The Reports should be provided to DSMC members at least one week prior to the date of the meeting.

14. Minutes of the DSMC Meeting

Two sets of minutes will be prepared from the meeting: Open Minutes and Closed Minutes. The open minutes will be prepared by the trial project manager. These will describe the proceedings in the Open Session of the DSMC meeting and will summarise the recommendations by the DSMC.

The DSMC will keep minutes of their closed session of the meeting. As it is likely that these minutes could contain unblinded information, it is important that they are not made available to anyone outside the DSMC. Copies will be archived by the DSMC Chair, for distribution to the lead investigators, and regulatory authorities at the time of study closure, if requested.

15. Recommendations to the Management Committee (MC)

At each meeting of the DSMC during the conduct of the trial, the DSMC will make a recommendation to the ARISE FLUIDS trial Management Committee. This recommendation will be based primarily on safety and efficacy considerations and will be guided by statistical monitoring guidelines defined in this Charter.

Possible recommendations from the DSMC include:

- Trial to continue as planned
- Trial to continue with modification/s
- Early stopping due to safety
- Proposing or commenting on proposed changes to protocol.

This recommendation will be communicated to the trial chief investigators in writing.

The ARISE FLUIDS trial Management Committee is jointly responsible with the DSMC for safeguarding the interests of participating patients and for the conduct of the trial. The ARISE FLUIDS trial Management Committee will be ultimately responsible for deciding whether to continue, modify or to stop the trial based on the DSMC recommendations. The ARISE FLUIDS Management Committee will communicate to site investigators and applicable ethics committees the DSMC recommendations.

The DSMC will be notified of all protocol amendments as detailed in section 2.

16. Statistical Monitoring Guidelines

Stopping rules for efficacy will be based on the HayBittle-Peto boundary approach with a p-value of 0.005 used to indicate benefit at interim whilst retaining a p-value of 0.05 for final analysis.

There are no predefined stopping rules for aspects pertaining to safety. As foremost experts in this field, the DSMB can recommend stopping the trial for safety aspects based on whatever information (both internal and external to the trial) they deem relevant. This trial will not be stopped for futility.

DSMC Member	Signature	Date
[redacted]	[redacted]	[redacted]
[redacted]	[redacted]	[redacted]
[redacted]	[redacted]	[redacted]
[redacted]	[redacted]	[redacted]
[redacted]	[redacted]	[redacted]

The DSMC member signature on this charter or email approval indicates approval of the content and agreement to adhere to the charter.

Figure 1.

ARISE FLUIDS trial organisational structure diagram

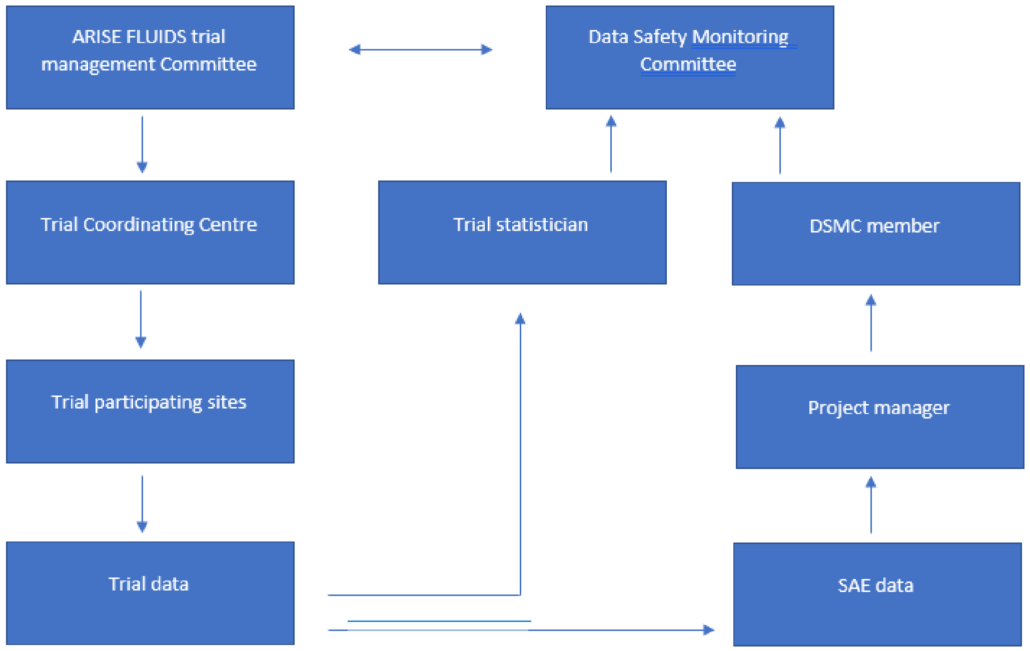

Supplement: online supplemental file 5 [file bmjopen-15-7-s005.pdf]
