# Supplementary material for: Australasian Resuscitation In Sepsis Evaluation: FLUid or vasopressors In emergency Department Sepsis (ARISE FLUIDS) trial: study protocol
Source: BMJ Open. 2025 Jul 20;15(7):e101215. doi: 10.1136/bmjopen-2025-101215 (PMC12278162; doi:10.1136/bmjopen-2025-101215)
Supplement: online supplemental file 6 [file bmjopen-15-7-s006.pdf]

Participant Information Sheet/Consent Form
Consent to Continue Participation

Interventional Study – *Adult providing own consent (consent to continue)*

[Insert site name]

**Title**	Australasian Resuscitation In Sepsis Evaluation: FLUid or vasopressors In emergency Department Sepsis
**Short Title**	ARISE FLUIDS study
**Protocol Number**	ANZIC-RC/SP002
**Project Sponsor**	Monash University
**Coordinating Principal Investigator/ Principal Investigator**	*[Coordinating Principal Investigator/ Principal Investigator]*
**Associate Investigator(s)** *(if required by institution)*	*[Associate Investigator(s)]*
**Location**	*[Hospital name]*

Part 1 What does participation involve?

1 Introduction

[Introduction paragraph for VICTORIAN SITES]

You were enrolled in this research project in accordance with Victorian law.

For some trials a health practitioner is able to administer treatment as part of medical research in an emergency to a person without consent, as long as there is no advanced care directive that prohibits that treatment, and the practitioner believes on reasonable grounds that the treatment is urgent being necessary to:

- save the person's life; or
- prevent serious damage to the person's health; or
- prevent the person from suffering or continuing to suffer significant pain or distress.

As the participant, you are now invited to consider your continued participation in this research project.

[Introduction paragraph for WA SITES]

You have been enrolled in this research study in accordance with Western Australian law (Guardianship and Administration Amendment (Medical Research) Act 2020). As you were not able to consent to participate in the study at the time of the treatment, the study team provided consent on your behalf. This can only occur if there is no advanced care directive that prohibits the treatment and there is a determination from an Independent Medical Practitioner that the

research was in your best interests, or not adverse to your interests. We are now asking if you would continue to participate in the study.

[Introduction paragraph for NON-VICTORIAN NON-WA SITES]

As the participant, you are invited to consider your continued participation in the ARISE FLUIDS study. You were enrolled in this research project [in accordance with ***[insert relevant state law***] which allowed the research to proceed] when it was not possible to obtain consent in advance due to your condition at the time you were enrolled.

[All sites]

This Information Sheet/Consent Form tells you about the research project. It explains the tests and treatments involved. Knowing what is involved will help you decide if you want to continue to take part in the research.

Please read this information carefully. Ask questions about anything that you don't understand or want to know more about. Before deciding whether or not to continue to take part, you might want to talk about it with a relative, friend or local doctor.

Participation in this research is voluntary. If you don't wish to continue to take part, you don't have to. You have received and will continue to receive the best possible care whether or not you continue to take part.

If you decide you want to continue to take part in the research project, you will be asked to sign the consent section. By signing it you are telling us that you:

- Understand what you have read
- Consent to continuing to take part in the research project
- Consent to the tests and treatments that are described
- Consent to the use of your personal and health information as described.

You will be given a copy of this Participant Information and Consent Form to keep.

2 What is the purpose of this research?

You were enrolled in this study because you required urgent treatment for your septic shock in the Emergency Department (ED). Septic shock is a serious illness where the body's response to an infection injures its own tissues through a process which is known as inflammation. This can affect important organs in the body such as the heart, kidneys and lungs. The key feature of septic shock is abnormally low blood pressure which is insufficient to supply blood to the tissues. It is very important that this is treated immediately.

This project is comparing two treatment strategies for patients with septic shock. Both of these treatment strategies are part of the usual or standard care treatment of patients with septic shock. Currently doctors use both these strategies, or a variation of them, however there is no agreement amongst doctors around the world about which is best. The doctors and the team associated with this research project are dedicated to giving patients the best possible treatments. The aim of this study is to compare these 2 standard care strategies to see which may be better to help keep patients alive and out of hospital.

Both treatment strategies involve fluids and medications (known as vasopressors) given into the vein both of which support the blood pressure and help keep the body organs working properly. One strategy gives less fluid and starts vasopressor medication to support the blood pressure and body organs ('vasopressors' arm). The other strategy involves more fluid initially before a later start of the medication (if required) to support the blood pressure and body organs ('fluids' arm).

Medications, drugs and devices have to be approved for use by the Australian Federal Government. All the fluids and medications used in this study are approved in Australia. This means they are not experimental treatments. Both the ‘fluids’ and ‘vasopressors’ treatment approaches are within the spectrum of accepted usual care. The treating team will enrol a patient into the research only if they are uncertain about which strategy is the right one for that particular patient.

This research is endorsed by the Australasian College for Emergency Medicine Clinical Trials Network and the Australian and New Zealand Intensive Care Society Clinical Trials Group. It is being conducted by Monash University on behalf of the investigators.

3 What does participation in this research involve?

Participation in this research involves the following:

<u>In the Emergency Department</u>

This study is a randomised controlled trial. You had an equal (50/50) chance of receiving either of the above treatments. Sometimes we do not know which treatment is best. To find out we need to compare different treatments. We put people into groups and give each group a different treatment. The results are compared to see if one is better. To try to make sure the groups are the same, each participant is put into a group by chance (random). Neither the treating team or you can control which of the two treatment arms was assigned.

The treating doctor assessed you as being eligible to be in this research study and you were randomly assigned, like the flip of a coin, to receive treatment with either:

- **Vasopressors** i.e. less fluids and early start of blood support medication <u>OR</u>
- **Fluids** i.e. more fluids and later start of blood support medication, if required.

You would have received treatment for your septic shock if required for a minimum of 6 hours. If your condition required you to be admitted to the intensive care unit (ICU) for further monitoring then you may have received study treatment for up to a maximum of 24 hours. Your treating doctor decided on the type of fluid and the type of blood support medications received. The treating staff continually monitored you for your fluid status and blood pressure support required. This is a part of usual care for patients in the ED/ICU who have septic shock. You received all other standard care as determined by the treating doctor. No additional tests, examinations or samples of blood were or will be required for this research study.
Once the study treatment period was completed (either 6 or up to a maximum of 24 hours) all treatment, including for your septic shock, would have been the treating doctor’s decision.

You would have received the fluids through a small plastic tube inserted in a vein (intravenous catheter or ‘drip’). The blood pressure support medication may have been given through a small plastic tube inserted in a vein (intravenous catheter or ‘drip’) or a drip into a large vein near the patient’s collarbone known as a central venous catheter (CVC). Both of these are a part of standard care administration of fluids and blood pressure support medication in the ED/ICU.

The timing of this consent discussion may vary but it is anticipated that all of the above procedures will be completed. The study investigator or research coordinator will be able to advise you of this.

<u>Following discharge from hospital</u>

Either you or if you prefer a relative/friend you nominate will be contacted by a research co-ordinator/nurse from the hospital. This telephone call will occur at approximately 3 months after participation started in this research study. This phone call is to determine how well you are recovering and should take no longer than 10 minutes. You, or your nominated relative/friend, will also be asked if you have been admitted to and then discharged from any other hospitals,

rehabilitation hospitals or nursing home in this 3 month period. You may be given a diary/information collection sheet to take home to help you keep track of this information so you will have it ready for the 3 month phone call.

At approximately 6 months and again at 12 months after participation started in this research study a phone call will be made to you or your relative/friend to collect information on your recovery. The research coordinator/nurse from your hospital will collect information about your quality of life i.e. able to walk, look after yourselves and generally how you are going. It is expected this phone call will take about 10-15 minutes. In order for this phone call to take place, we will ask you to supply contact phone numbers for this phone call. The questions may be mailed or emailed a few weeks before the phone call to save your time during the call.

In addition, the researchers would like to have access to your medical record to obtain information relevant to the study. The researchers may also contact the other hospitals, rehabilitation hospitals or nursing homes, if you mention any in the 3 month phone call, to verify the admission and discharge dates. This will happen only if required as this is essential information for measuring the effectiveness of each of the study treatments.

There are no additional costs associated with participating in this research project, nor will you be paid. All medication, tests and medical care you required as part of the research project will have been provided to you free of charge.

If you decide to continue participation in this research project, the study doctor may inform your local doctor.

This research project has been designed to make sure the researchers interpret the results in a fair and appropriate way and avoids study doctors or participants making incorrect conclusions.

Your general hospital care was/will not be affected in any other way by this research study.

4 What do I have to do?

You would have received all study treatment while in the ED or the ICU, if your condition required ICU admission. No additional tests, examinations or samples of blood were or will be required for this research study. You, or if you prefer a relative/friend you nominate, will be contacted at 3, 6 and 12 months after the study to answer questions as detailed above.

5 Other relevant information about the research project

This research study will be conducted at approximately 50 hospitals primarily in Australia and New Zealand and enrol 1000 patients over 3 years.

6 Do I have to take part in this research project?

Participation in any research project is voluntary. If you do not wish to take part, you do not have to. If you decide to continue to take part and later change your mind, you are free to withdraw from the project at any stage.
If you do decide to take part, you will be given this Information and Consent Form to sign and you will be given a copy to keep.

Your decision whether to continue to take part or not, or to take part and then withdraw, will not affect your routine treatment, relationship with those treating you or your relationship with *[Institution]*.

7 What are the alternatives to participation?

You do not have to continue to take part in this research project to receive treatment at this hospital. Other options are available; these include not to continue participation and receive no follow up phone calls or collection of data. Your study doctor will discuss this with you before you decide whether or not to continue involvement in this research project. You can also discuss the options with your local doctor.

8 What are the possible benefits of taking part?

We cannot guarantee or promise that you will receive any benefits from this research. This study aims to further medical knowledge and may improve future treatment of patients who come to the Emergency Department with septic shock. There may be no clear benefit to you from this research.

9 What are the possible risks and disadvantages of taking part?

Medical treatments often cause side effects. You may have had none, some or all of the effects listed below, and they may have been mild, moderate or severe. If you had any of these side effects, or are worried about them, talk with the doctor. Your doctor would also have been looking out for side effects.

Septic shock is a serious illness and patients may experience severe complications including death as a result of this illness. The objective of this trial is to determine if this risk can be reduced.

Because the trial is comparing two common accepted treatments, we do not expect that you were exposed to any greater risk than if they were not in the trial. There may be side effects that the researchers do not expect or do not know about that may be serious. Tell your study doctor immediately about any new or unusual symptoms.

Many side effects go away shortly after treatment ends. However, sometimes side effects can be serious, long lasting or permanent. If a severe side effect or reaction occurred, the study doctor may have stopped treatment. The study doctor will discuss the best way of managing any side effects.

There are known risks associated with the fluids administered and the blood support medications used for patients with septic shock. As these are all commonly used fluids and medications, the doctors and nurses who were caring for you while in the ED/ICU are trained to recognise the risks associated with them and treat accordingly. Any unexpected complications would have been recorded as part of the routine safety monitoring of the trial.

These are:

<u>Central Venous Catheter (CVC) complications</u>
CVC complications are uncommon. They may include: infection, injury to the blood vessel causing bruising and/ or bleeding or a blood clot inside the blood vessel (thrombosis) and puncture of the lung, causing collection of air into the chest. These complications are treatable and all patients will have been assessed for the presence of any of these complications.

<u>IV Fluid administration complications</u>
Giving fluid into a vein can cause fluid overload which can impair the function of organs such as the lungs and kidneys. This is reversible by slowing the speed at which the fluid is given, giving less fluid and sometimes by giving other medications. Sometimes it may mean that patients require assistance with breathing with oxygen.

<u>Blood pressure support medication complications</u>
Medications given to support the blood pressure can cause abnormal heart rhythm or rarely, a decreased blood supply to the heart and limbs. Vasopressor medication can leak into the tissues

and in very rare cases this can lead to skin and tissue damage. Patients receiving vasopressors are routinely monitored for such complications as part of routine practice.

All patients in the ED/ICU have continuous monitoring of their blood pressure and vital signs. Significant changes to your vital signs would have been detected and treated early.

10 What will happen to my test samples?

This study involves no additional tests, examinations or samples of blood. Routine blood test results, if available, may be used for this study.

11 What if new information arises during this research project?

Sometimes during the course of a research project, new information becomes available about the treatment that is being studied. If this happens, your study doctor will tell you about it and discuss with you whether you want to continue in the research project. If you decide to withdraw, your regular health care will continue. However as all study treatments have finished, this is unlikely to occur or have any impact.

12 Can I have other treatments during this research project?

You would have received all other standard care treatments as needed while in the ED/ICU.

13 What if I withdraw from this research project?

If you decide to withdraw from the project, please notify a member of the research team before you withdraw. This notice will allow that person or the research supervisor to discuss any health risks or special requirements linked to withdrawing.

If you do withdraw consent during the research project, the study doctor and relevant study staff will not collect additional personal information about you, although personal information already collected will be retained to ensure that the results of the research project can be measured properly and to comply with law. You should be aware that data collected by the research staff up to the time you withdraw will form part of the research project results. If you do not want them to do this, you must tell them.

14 Could this research project be stopped unexpectedly?

The project may be stopped unexpectedly for a variety of reasons.
These may include reasons such as:

- Unacceptable side effects
- Either of the treatments being shown not to be effective
- Either of the treatments being shown to work and not need further testing

15 What happens when the research project ends?

Once the 12-month follow up telephone call is completed, your participation in the study will be finished.

Part 2 How is the research project being conducted?

16 What will happen to information about me?

By signing the consent form you consent to the study doctor and relevant research staff collecting and using personal information about you for the research project. Any information obtained in connection with this research project that can identify you will remain confidential. Only the research team at the hospital will know your identity. All study information will be kept in a locked filing cabinet or on a password protected computer in the secure research office at *[hospital name]* which is only accessible to the research staff. The research study results will be held securely at the coordinating centre at Monash University. The research information will be kept for 15 years then will be securely deleted and shredded.

Please be advised that your information will be used for the purpose of this research project, as well as in future ethically approved research projects that are an extension of, or closely related to, the aims of this study. Your information will only be disclosed with permission, except as required by law.

The data collected in this study will be analysed at Monash University in Melbourne, Victoria, by members of the project research team.

Information about you may be obtained from your health records held at this and other health services for the purpose of this research. By signing the consent form you agree to the study team accessing health records only if they are relevant to your participation in this research project.

Your health records and any information obtained during the research project are subject to inspection (for the purpose of verifying the procedures and the data) by the relevant authorities and authorised representatives of the Sponsor, Monash University, the institution relevant to this Information Sheet, *[Name of institution]*, or as required by law. By signing the Consent Form, you authorise release of, or access to, this confidential information to the relevant study personnel and regulatory authorities as noted above.

It is anticipated that the results of this research project will be published and/or presented in a variety of forums. In any publication and/or presentation, information will be provided in such a way that you cannot be identified, except with permission.

Information about your participation in this research project may be recorded in your health records.

In accordance with relevant Australian *and/or [Name of state/territory]* privacy and other relevant laws, you have the right to request access to your information collected and stored by the research team. You also have the right to request that any information with which you disagree be corrected. Please contact the study team member named at the end of this document if you would like to access your information.

Any information obtained for the purpose of this research project and for the future research described in Section 16 that can identify you will be treated as confidential and securely stored. It will be disclosed only with your permission, or as required by law.

17 Complaints and Compensation

If you suffer any injuries or complications as a result of this research project, you should contact the study team as soon as possible and you will be assisted with arranging appropriate medical treatment. If you are eligible for Medicare, you can receive any medical treatment required to treat the injury or complication, free of charge, as a public patient in any Australian public hospital.

18 Who is organising and funding the research?

This research has been initiated by a group of investigators in Australia and New Zealand. This research is being conducted by Monash University on behalf of these investigators.

This study is funded by the Australian Government and has received a grant through the Medical Research Future Fund.

No member of the research team will receive a personal financial benefit from your involvement in this research project (other than their ordinary wages).

19 Who has reviewed the research project?

All research in Australia involving humans is reviewed by an independent group of people called a Human Research Ethics Committee (HREC). The ethical aspects of this research project have been approved by the HREC of North Sydney Local Health District. Approval for this study has been given by *[Name of institution]*, where the research will be carried out.

This project will be carried out according to the *National Statement on Ethical Conduct in Human Research (2007)-Updated 2018*. This statement has been developed to protect the interests of people who agree to participate in human research studies.

20 Further information and who to contact

The person you may need to contact will depend on the nature of your query.

If you want any further information concerning this project or if you have any medical problems which may be related to your involvement in the project (for example, any side effects), you can contact the principal study doctor on *[phone number]* or any of the following people:

Clinical contact person/s

Name	*[Name]*
Position	*[Position]*
Telephone	*[Phone number]*
Email	*[Email address]*

For matters relating to research at the site at which you are participating, the details of the local site complaints person are:

Complaints contact person

Name	*[Name]*
Position	*[Position]*
Telephone	*[Phone number]*
Email	*[Email address]*

If you have any complaints about any aspect of the project, the way it is being conducted or any questions about being a research participant in general, then you may contact:

Reviewing HREC approving this research and HREC Executive Officer details

Reviewing HREC name	North Sydney Local Health District HREC
HREC Executive Officer	Executive Officer
Telephone	02 9906 4590
Email	NSLHD-Research@health.nsw.gov.au

Local HREC Office contact (Single Site -Research Governance Officer)

Name	*[Name]*
Position	*[Position]*
Telephone	*[Phone number]*
Email	*[Email address]*

Consent Form- *Adult providing own consent*

**Title**	Australasian Resuscitation In Sepsis Evaluation: FLUid or vasopressors In emergency Department Sepsis
**Short Title**	ARISE FLUIDS study
**Protocol Number**	ANZIC-RC/SP002
**Project Sponsor**	Monash University
**Coordinating Principal Investigator/ Principal Investigator**	*[Coordinating Principal Investigator/ Principal Investigator]*
**Associate Investigator(s)** *(if required by institution)*	*[Associate Investigator(s)]*
**Location** *(where CPI/PI will recruit)*	*[Location where the research will be conducted]*

Declaration by Participant

I have read the Participant Information Sheet or someone has read it to me in a language that I understand.

I understand the purposes, procedures and risks of the research described in the project.

I give permission for my doctors, other health professionals, hospitals or laboratories outside this hospital to release information to *[Name of Institution]* concerning my disease and treatment for the purposes of this project. I understand that such information will remain confidential.

I have had an opportunity to ask questions and I am satisfied with the answers I have received.

I freely agree to continue participation in this research project as described and understand that I am free to withdraw at any time during the study without affecting my future health care.

I understand that I will be given a signed copy of this document to keep.

Name of Participant (please print) ______________________________

Signature ______________________ Date ______________________

Declaration for participant: unable to read the information and consent form or telephone consent

Name of Witness* to Participant's Signature/consent (please print) ______________________________

Signature ______________________ Date ______________________

* Witness is not to be the investigator, a member of the study team or their delegate. In the event that an interpreter is used, the interpreter may not act as a witness to the consent process. Witness must be 18 years or older.

Declaration by Study Doctor/Senior Researcher†

I have given a verbal explanation of the research project, its procedures and risks and I believe that the participant has understood that explanation.

Name of Study Doctor/ Senior Researcher† (please print) ______________________________

Signature ______________________ Date ______________________

† A senior member of the research team must provide the explanation of, and information concerning, the research project.

Note: All parties signing the consent section must date their own signature.
